# Polymer-Engineered Ionogels for Lithium-Based Energy Storage: Interactions, Processing, and Electrode Interfaces

**DOI:** 10.3390/polym18212737

**Published:** 2026-11-03

**Authors:** Seung-Chan Jung, Myeong-Won Kang, Byeongji Beom, Wonjun Jang, Jiyun Noh, Deepak Dubal, Jae-Hee Han

**Affiliations:** 1 Department of Materials Science and Engineering, Gachon University, Seongnam 13120, Republic of Korea; jsc4486@gachon.ac.kr (S.-C.J.); james1129@gachon.ac.kr (M.-W.K.); jjkk3239@gachon.ac.kr (B.B.); juni1205@gachon.ac.kr (W.J.); 2 Department of Chemical Engineering and Materials Science, Ewha Womans University, Seoul 03760, Republic of Korea; shdmltk@ewha.ac.kr; 3 Centre for Materials Science, School of Chemistry and Physics, Queensland University of Technology, Brisbane, QLD 4000, Australia; deepak.dubal@qut.edu.au

**Keywords:** ionogel, ionic liquid, polymer electrolyte, polymer–ionic liquid interaction, ion transport, electrode–electrolyte interface

## Abstract

By immobilizing ionic liquids (ILs) within polymer networks, ionogels offer a route to quasi-solid-state electrolytes with liquid-like ion mobility and solid-like dimensional stability. Their practical performance, however, is not determined by IL content or total ionic conductivity alone. Frequently reported limitations include low Li^+^ transference numbers, polymer–IL demixing, creep, insufficient electrode wetting, and unstable interphases. This review develops a mechanistically informed framework that traces how polymer chemistry and polymer–IL–lithium salt interactions shape process-induced microstructures, which in turn influence ion transport, mechanical response, and electrode interfaces. We examine electrostatic interactions, hydrogen bonding, and competitive Li^+^ coordination across representative polymer–IL systems, and compare fabrication routes in terms of the confinement, phase connectivity, and electrode contact they generate. The routes considered include solution casting, phase inversion and scaffold impregnation, electrospinning, thermal or photoinitiated in situ polymerization, physical self-assembly, interpenetrating-network and multiple-network formation, hybrid sol–gel processing, and thin-film patterning. The review distinguishes structural evidence from Li^+^-specific transport evidence and considers differences in measurement and cell conditions. Load-bearing networks and continuous ion-rich domains can mitigate the transport–mechanical trade-off, but their benefits require verification of Li^+^ transport and interfacial stability under relevant operating conditions. Minimum reporting criteria for meaningful comparison and scale-up are proposed.

## 1. Introduction

The discovery of poly(ethylene oxide) (PEO)–alkali metal salt complexes in the 1970s established the concept of polymer electrolytes, demonstrating that polymer chains can solvate and transport ions [1]. Polymer electrolytes can limit leakage and enable thin-film processing, while their interfacial conformability depends on composition and fabrication history. However, conventional solvent-free PEO-based electrolytes generally suffer from insufficient ionic conductivity at room temperature because of the high crystallinity of PEO and the restricted segmental motion of the polymer chains. In contrast, gel polymer electrolytes (GPEs) containing organic solvents can provide high ionic conductivity, but concerns remain regarding solvent volatility, flammability, solvent loss, and long-term dimensional stability.

Ionic liquids (ILs) are generally defined as salts that are liquid below 100 °C and offer distinct advantages, including low vapor pressure, high thermal stability, and a broad compositional design space [2,3,4]. When ILs are immobilized within polymeric or inorganic networks, ionogels can be formed, effectively suppressing liquid leakage [5,6,7]. However, because ionogels typically contain a substantial fraction of liquid-phase ILs, they should be distinguished from strictly defined “all-solid-state” electrolytes. Here, “quasi-solid-state electrolyte” denotes an electrolyte containing an immobilized liquid phase, whereas “solid-like” describes its macroscopic mechanical behavior [8,9,10].

**Figure 1:**
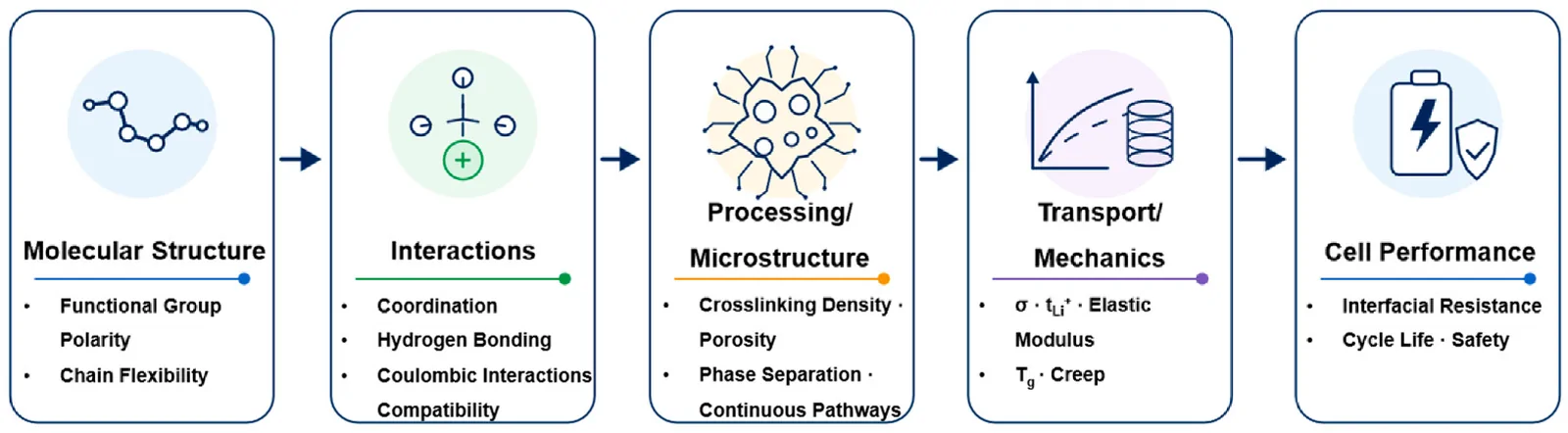
Mechanism-informed design framework for polymer-based ionogel electrolytes. This conceptual synthesis links polymer chemistry and molecular interactions to process-induced microstructures, ion transport and mechanical response, and cell behavior. The arrows indicate proposed relationships rather than causal links established in every system. Interfacial reconstruction and feedback are discussed in Section 6.

Chen et al. reviewed ionogel host matrices, confinement strategies, and their implications for electrolyte properties and lithium-battery applications [8]. Ma et al. examined IL/PIL-based liquid, quasi-solid, and solid electrolytes, including ion-coordination and transport mechanisms [10]. More recently, Zhao et al. presented a structure–property–function framework for multifunctional ionogels in flexible energy-storage devices [11]. Building on these contributions, the present review focuses on polymer-based ionogels for lithium-ion and lithium-metal batteries and examines how polymer–IL–lithium salt interactions and fabrication-induced microstructures jointly influence ion transport, mechanical integrity, and electrode-interface behavior. Particular attention is given to distinguishing total ionic conductivity from Li^+^-specific transport evidence, structural correlations from mechanistic support, and material-design effects from differences in measurement and cell conditions. Polymers that are highly miscible with ILs can yield homogeneous films and high electrolyte uptake, but excessive plasticization may lead to increased creep. In contrast, IL-insoluble domains can serve as physical crosslinking points but may also disrupt or block ion-conduction pathways. Therefore, guided by the mechanism-informed design framework illustrated in Figure 1, this review organizes the literature along a mechanistic sequence linking polymer–IL–lithium salt interactions, process-induced microstructures, ion transport and mechanical response, and electrode-interface behavior. It thereby shifts the emphasis from cataloguing material combinations to explaining why particular combinations of composition and processing generate specific structures and ion-transport behavior. The review also evaluates the experimental conditions required to interpret reported performance and proposes minimum characterization and reporting criteria for mechanistic comparison and scale-up.

## 2. Fundamentals of Polymer–Ionic Liquid–Lithium Salt Interactions

### 2.1. Polymer–Ionic Liquid Compatibility: Thermodynamic and Molecular Factors

Ionic liquids are liquids composed of cations and anions held together by strong Coulombic interactions. Polymer dissolution or swelling in an IL depends on the free-energy balance between polymer–ion interactions, the cohesion of the original components, and the entropy of mixing [12,13]. Therefore, miscibility cannot be reliably predicted solely on the basis of dielectric constant or simple “polarity matching.” The nature of the functional groups, polymer-chain flexibility, cation alkyl-chain length, degree of charge delocalization in the anion, water content, and salt concentration can all simultaneously alter the free energy of mixing.

Ether, carbonyl, and nitrile groups provide distinct ion-interaction environments. Kumar et al. examined ion–polymer interactions in IL-plasticized PEO electrolytes [14]. The carbonyl groups of poly(methyl methacrylate) (PMMA) provide potential Li^+^ coordination sites, although the matrix may remain relatively glassy; PMMA-containing blends include the porous PVDF-HFP/PMMA system studied by Zhai et al. [15]. The polar nitrile groups of polyacrylonitrile (PAN) influence electrolyte affinity, and Rao et al. investigated an electrospun PAN/PMMA scaffold [16]. Poly(vinylidene fluoride-co-hexafluoropropylene) (PVDF-HFP) provides a processable fluorinated scaffold for porous membranes. Cheruvally et al. used interconnected electrospun PVDF-HFP membranes to incorporate IL-based electrolytes [17]. In these porous systems, electrolyte-filled pathways and scaffold morphology must be considered alongside polymer chemistry when interpreting ionic conductivity; electrolyte uptake alone does not directly establish altered Li^+^ coordination.

### 2.2. Polymer–Ionic Liquid–Lithium Salt Interaction Mechanisms

The principal interaction modes and their implications for electrolyte design are summarized in Table 1.

These interactions do not operate independently. For example, acrylic acid-based networks containing COOH groups can provide hydrogen-bonding and potential Li^+^ coordination sites, while partial incompatibility with the IL may induce phase separation [23,24]. In the ETPTA–MPC ionogel studied by Li et al., FTIR, Raman, and NMR analyses, together with DFT binding-energy calculations, support composition-dependent changes in Li^+^ coordination with MPC and bis(trifluoromethanesulfonyl)imide (TFSI^−^). XPS depth profiling separately revealed a LiF-rich solid electrolyte interphase (SEI) after cycling [22]. These structural, computational, and surface-chemical observations should not be equated with direct measurements of ligand-exchange or interfacial desolvation rates. In PILs, fixed ionic groups can promote affinity with an IL, whereas ionic aggregation can restrict mobility [18,19,20,30,31]. The consequences depend on whether cationic or anionic groups are tethered; PIL formation alone does not establish single-Li^+^-ion conduction.

### 2.3. Li^+^ Solvation and Effective Charge Carriers

In TFSI^−^ or bis(fluorosulfonyl)imide (FSI^−^)-based IL-rich electrolytes, Li^+^ often coordinates with anion oxygen atoms, although polymer donors and additives can modify this coordination environment [21]. Because the total ionic conductivity, σ, reflects the collective motion and correlations of Li^+^, IL cations, anions, and associated ionic species, it does not directly quantify Li^+^ transport. Reported Li^+^ transference numbers should therefore be interpreted with their measurement definitions, reference frames, and experimental conditions [32]. The PC-containing formulation discussed in Section 3.3 provides a specific example [33]. Anion motion and a limited Li^+^ contribution can contribute to concentration polarization under current, but the resulting concentration and field distributions also depend on cell geometry and operating conditions.

Polymer functional groups and phase organization can modify ion transport through three main routes. First, ether oxygen atoms or carbonyl groups can provide alternative Li^+^ coordination sites and modify Li^+^–anion association [14,21]. Second, fixed anionic groups can restrict anion motion, depending on the polymer charge architecture [34]. Third, phase separation can modify the connectivity and orientation of ion-rich domains, thereby changing macroscopic ion-transport pathways [23,27,28,35]. Stronger Li^+^–polymer binding does not necessarily improve transport, because ion motion also depends on segmental relaxation and coordination-site exchange. A design objective is therefore to balance Li^+^ solvation with sufficiently rapid coordination exchange, rather than simply maximize binding strength.

## 3. Component Design of Polymer-Based Ionogels

### 3.1. Polymer Matrix

PEO and poly(ethylene glycol) (PEG)-based systems offer favorable Li^+^ coordination and low glass transition temperatures (T_g_), but their practical performance is limited by crystallinity, oxidative instability at high voltages, and insufficient interfacial stability against Li metal. Crystallinity can be reduced through crosslinking, side-chain engineering, or IL incorporation; however, excessive crosslinking density can suppress chain relaxation and thereby decrease ionic conductivity [1,14]. Acrylate and methacrylate precursors enable photo- or thermally initiated network formation, while the resulting carbonyl-containing matrices provide Li^+^ coordination sites. Their composition and crosslink density can be adjusted to balance IL compatibility and mechanical stability. Accordingly, PEGDA [29,36], ETPTA [22], BA combined with EGDMA [33], and acrylic acid [23,24] have been used as network-forming precursors for ionogels.

PVDF-HFP is suited to porous supports when electrolyte uptake, film integrity, and processability are priorities [15,16,17,37,38]. Its performance should therefore be assessed through pore connectivity, IL retention, and through-plane resistance rather than polymer chemistry alone. In such scaffold-based systems, ionic conduction can be dominated by the IL-rich phase within the pores rather than transport through the polymer bulk. PAN/PMMA provides another electrospun scaffold option [16]. PIL selection should account for fixed-charge architecture, counterion chemistry, and affinity for the selected IL [18,19,20]. Representative implementations include IL-rich free-standing gels [31], PIL block-copolymer films [30], and composite [38], silica-hybrid [39], or self-healing [40] electrolytes. Block copolymers can spatially segregate IL-phobic blocks as physical crosslinking domains and IL-philic blocks as ion-rich phases [28].

### 3.2. Ionic Liquid Cations and Anions

Imidazolium-based ILs can provide favorable ionic conductivity and participate in hydrogen-bonding interactions with suitable polymer functional groups. Their viscosity, polymer interactions, and reductive stability depend on the cation substituents, anion, lithium salt concentration, and test conditions [3,4]. Pyrrolidinium-based formulations are often selected for lithium-metal systems because of their favorable reductive stability, although viscosity and the measured stability window remain dependent on the anion, salt concentration, and test conditions [3,4,33,41,42,43]. Phosphonium-based ILs have also been incorporated into acrylic-acid-based ionogels [23,24]. These examples illustrate formulation choices rather than a universal ranking of cation families.

The delocalized charge of TFSI^−^ is relevant to salt dissociation, but Li^+^–anion association remains dependent on electrolyte composition. Its decomposition can generate LiF and N- and S-containing species [21]. Their presence alone does not establish a protective interphase; the interfacial function of these products is discussed in Section 6. FSI^−^-based formulations can offer low viscosity and favorable Li^+^ transport, but their thermal stability and interfacial reactivity must be evaluated for the specific electrolyte composition. For BF_4_^−^ and PF_6_^−^, hydrolysis and the formation of fluoride-containing by-products must be taken into consideration. Therefore, a “wide electrochemical stability window” should not be evaluated solely from linear sweep voltammetry (LSV) of the neat IL, but rather under conditions that account for the actual electrode material, electrode surface area, water content, and scan rate [3,4,21].

### 3.3. Lithium Salt Concentration and Additional Components

Increasing the lithium salt concentration raises the total Li^+^ concentration but does not necessarily increase the population of effectively mobile Li^+^ species, because increased viscosity and ionic association can offset the benefit of higher salt content. The optimum concentration therefore depends on the type of IL, the availability of coordination sites in the polymer, and the presence of immobilized anionic species. The effects of molecular cosolvents are likewise formulation dependent. Zhou et al. reported improved ionic conductivity and Li^+^ transference number upon propylene carbonate (PC) incorporation into a BA/EGDMA-based pyrrolidinium ionogel [33]. This example should not be interpreted as a universal benefit of molecular cosolvents. Their addition also changes the electrolyte composition, so the low volatility of the IL alone cannot establish the safety of the resulting mixture. Accordingly, the solvent content, volatility, and flammability of mixed-electrolyte ionogels should be explicitly reported and evaluated.

## 4. Fabrication Strategies: Process-Induced Microstructures

**Figure 2:**
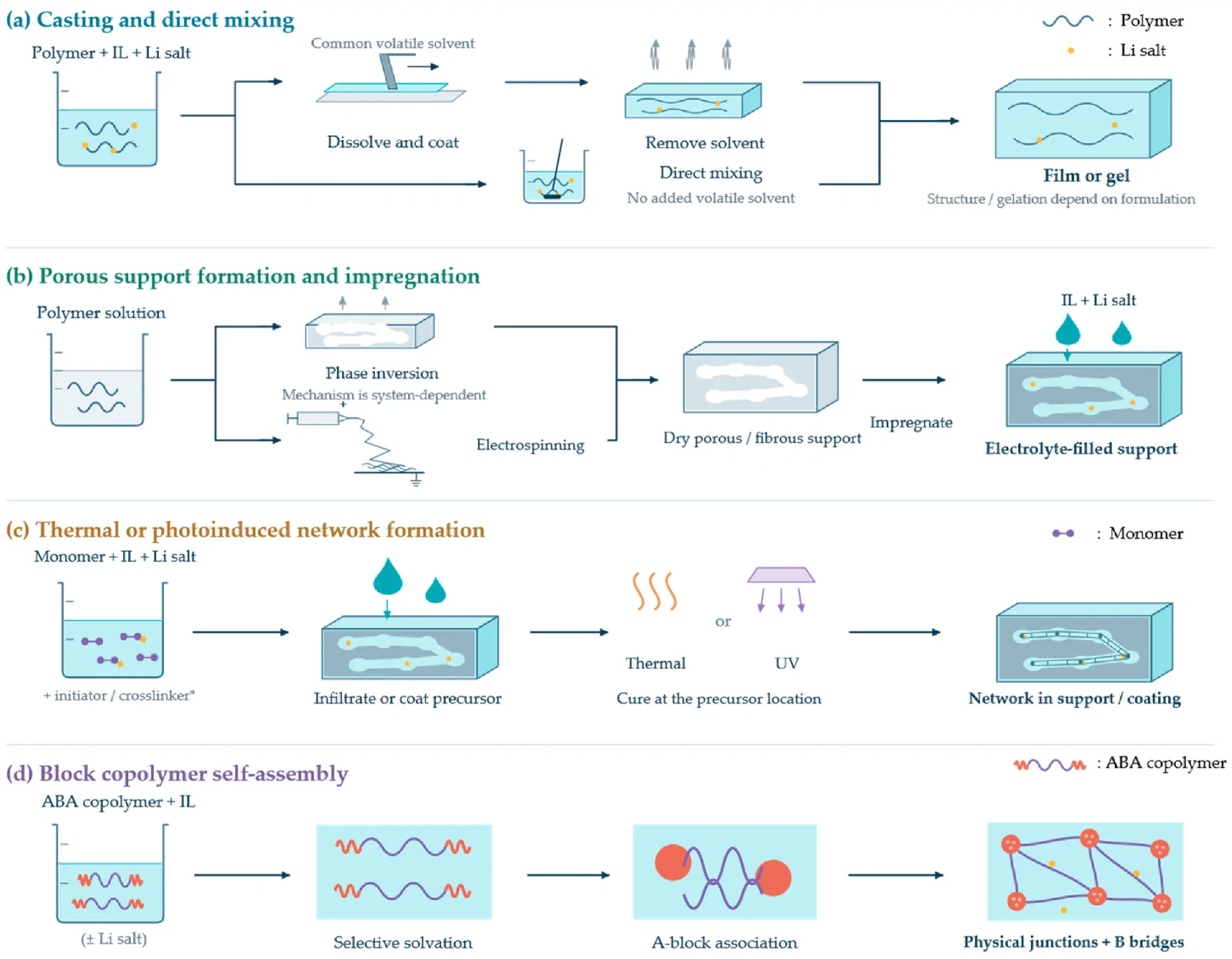
Schematic illustrations of fabrication routes and possible resulting structures of polymer-based ionogels. (**a**) Solution casting involves dissolution, coating, and removal of a common volatile solvent, whereas direct mixing proceeds without an added volatile solvent. The final morphology and gelation depend on the formulation. (**b**) Phase inversion or electrospinning produces a porous or fibrous support before impregnation with an IL/lithium-salt electrolyte. The phase-inversion mechanism is system-dependent. (**c**) Infiltration or coating of a reactive precursor followed by thermal or UV curing forms a network within a support or coating. (**d**) Selective solvation of an ABA block copolymer promotes association of the A end blocks into physical junctions connected by B-midblock bridges. The illustrations represent conceptual processing sequences and possible structures rather than microscopy images or experimentally established transport pathways. Through-plane phase continuity and Li^+^-specific transport require independent verification. IPNs, multiple crosslinking, and hybrid formation may accompany these routes (Section 4.5 and Section 4.6). * The use and identity of a crosslinker are formulation-dependent.

Figure 2 presents the inputs, main processing steps, and possible structures of four principal route families. Solvent removal, scaffold formation and filling, network curing, and phase self-assembly impose different controls on electrolyte distribution and dimensional stability. These routes can be combined: IPNs and multiple crosslinking are network-design strategies applicable across fabrication methods (Section 4.5), while hybrid formation and thin-film engineering introduce further processing choices (Section 4.6). A depicted ion-rich phase should not be treated as proof of through-plane continuity or Li^+^-specific transport.

### 4.1. Solution Casting and Direct Mixing

Solution casting forms films by dissolving the polymer, IL, and lithium salt in a common solvent, followed by coating and solvent removal. Safa et al. cast PDADMA-TFSI gels from acetone using 20 wt% PIL and 80 wt% of a 1 M LiTFSI/[EMIM][TFSI] electrolyte [31]. Ambient evaporation for 20 min was followed by vacuum drying at 90 °C for 72 h. Nykaza et al. instead cast a precursor block-copolymer film, exchanged its counteranion, and imbibed it with LiTFSI/[EMIM][TFSI] [30]. The final films contained 67 ± 5 wt% salt-containing electrolyte. Casting, post-treatment, and electrolyte incorporation therefore need separate descriptions. Drying-induced phase evolution, residual solvent and water, and IL migration can change the final morphology; an initially homogeneous solution does not establish a homogeneous final film.

Direct mixing avoids an added common volatile solvent, but requires compatible components, manageable viscosity, adequate salt dissolution, and controlled addition order, mixing time, and temperature. Incomplete homogenization can create local differences in IL concentration, Li^+^ coordination, and mechanical response. Mixing alone does not specify how a supporting network forms; any subsequent gelation or curing step should be identified. For both casting and mixing, film thickness, conditioning, residual water or solvent, phase morphology, and IL redistribution should accompany conductivity measurements. These variables become particularly important in thicker or larger-area films.

### 4.2. Porous Scaffolds: Phase Inversion, Electrospinning, and Post-Impregnation

Porous supports are formed before electrolyte impregnation, with pore generation depending on the phase-inversion route. In the PVDF-HFP/PMMA study of Zhai et al., differential evaporation of DMAC and ethylene glycol induced phase separation; MIL-60 used a 40:60 polymer mass ratio [15]. This example should be distinguished from immersion-bath non-solvent-induced phase separation (NIPS). Electrospinning provides a separate route to fibrous supports, including PVDF-HFP [17] and PAN/PMMA [16]. Rao et al. impregnated a dried PAN/PMMA scaffold for 2 h with 1 M LiTFSI in a 2:1 mass mixture of PYR_14_TFSI and PEGDME [16]. The scaffold had 86% porosity and a maximum uptake of 480% relative to dry membrane mass. Support formation and electrolyte loading can thus be adjusted separately, but initial uptake does not establish retention during prolonged compression, heating, or bending.

In these systems, electrolyte uptake alone does not provide a sufficient measure of scaffold performance. Pore connectivity, tortuosity, pore-size distribution, and through-plane continuity determine whether the impregnated IL-rich phase forms effective ion-transport pathways across the membrane. Increasing porosity can reduce transport resistance and improve electrolyte loading, but may also weaken the supporting framework and facilitate electrolyte displacement under compression. Conversely, smaller pores may favor capillary retention when the electrolyte wets the pore surfaces, whereas poor pore connectivity can limit through-plane conduction. Pore size, wettability, and connectivity should therefore be considered separately. Because ion transport in these membranes occurs predominantly through the impregnated liquid-rich phase rather than through the polymer scaffold itself, conductivity should be interpreted together with porosity, thickness, electrolyte uptake, dimensional stability, and electrolyte retention after mechanical or thermal treatment. Scaffold design therefore requires balancing continuous ion-conduction pathways against sufficient mechanical confinement of the IL-based electrolyte.

### 4.3. Thermal and Photoinitiated In Situ Polymerization

Network formation can occur within a preformed support, an electrode coating, or another precursor-filled component; the location should be stated rather than inferred from the term in situ. Huang et al. immersed porous PVDF-HFP in a MHBIm-TFSI/PEGDA-containing precursor for 2 h and then polymerized it at 70 °C for 12 h under Ar [44]. This demonstrates network formation within a support and does not, by itself, establish curing inside an assembled cell. The thermal exposure and precursor chemistry must be compatible with the component being filled.

However, the precursor must be chemically compatible with the electrode active materials, binders, and current collectors. Photocuring requires adequate light penetration, whereas thermal curing requires control of temperature exposure and heat-induced side reactions. Both routes require assessment of conversion, curing shrinkage, residual monomers, and initiator-derived species; oxygen inhibition should be considered where relevant to the radical chemistry. In practical fabrication, the spatial distributions of the degree of cure and crosslinking density can strongly influence both impedance and resistance to short-circuiting. Therefore, Fourier-transform infrared spectroscopy (FTIR) based conversion, gel fraction, extractable content, and through-thickness curing uniformity should be included among the minimum reporting parameters.

Photoinduced curing requires optical access to the intended reaction volume [45]. Igbaroola et al. prepared acrylic-acid ionogels using 5 min irradiation at 250 mW cm^−2^, followed by vacuum drying at 60 °C for 24 h [24]. The irradiation time is therefore only one part of preparation. Separately, Wei et al. wetted an LFP cathode with a solvate-IL precursor containing PEGDA and PETEA and formed an ionogel layer by UV irradiation without an additional separator [36]. This electrode-integrated configuration should be distinguished from curing a free-standing film or a porous support; the suitability of other high-voltage implementations remains formulation-specific [42].

### 4.4. Physical Gelation and Block Copolymer Self-Assembly

When IL-insoluble end blocks aggregate and IL-philic midblocks bridge the resulting domains, an ABA copolymer can form physical junctions without covalent crosslinking [28,46]. Discrete IL-phobic domains may reinforce a continuous ion-rich matrix, whereas disconnected IL-rich domains can restrict long-range transport. He et al. demonstrated gelation of poly(styrene-block-ethylene oxide-block-styrene) in [BMIM][PF_6_] at polymer contents as low as 5 wt% [46]. This establishes physical gelation rather than Li-cell performance. Reprocessability, relaxation, and resistance to high-temperature creep must be validated for the selected composition and operating conditions [47].

Block volume fraction, molecular weight, polymer–IL selectivity, and thermal history determine the domain structure. Mizuno et al. related thermally induced changes among ABA-copolymer morphologies to mechanical and conductivity responses using structural characterization [28]. Such observations connect processing history to morphology, but do not establish a universal optimum for Li electrolytes. Where lithium salt is added, its effect on self-assembly must also be assessed. The PIL diblock films studied by Nykaza et al., for example, were prepared by casting and subsequent electrolyte imbibition and were characterized by in-plane conductivity; they should not be equated with every ABA physical-gel architecture [30]. Morphology, directional conductivity, and temperature-dependent rheology are needed together to test transport continuity and network stability.

### 4.5. Multiple Crosslinking, Interpenetrating Networks, and Phase Separation

**Figure 3:**
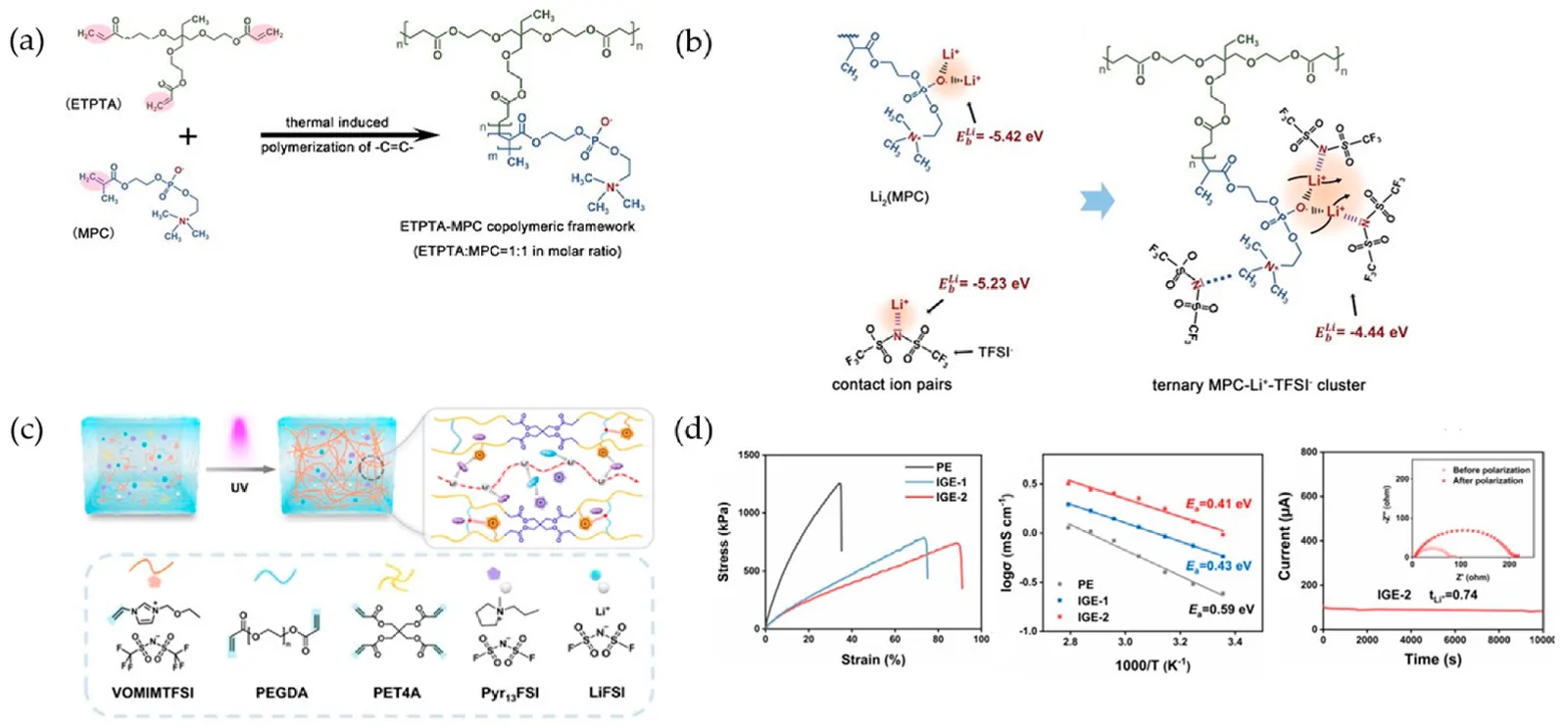
Schematics, calculations, and measurements relevant to ionogel network design. (**a**) Reaction schematic for thermal ETPTA−MPC copolymerization. (**b**) Coordination models and DFT−calculated Li^+^ binding energies for MPC/TFSI^−^ species; (**a**,**b**) adapted under CC BY 4.0 from the cited study [22]. (**c**) Schematic of UV−induced multiple−crosslinking network formation. (**d**) Experimental stress−strain curves, temperature-dependent total-conductivity plots, and DC polarization with an impedance inset; (**c**,**d**) adapted under CC BY 3.0 from the cited study [29]. The polarization panel retains the source Figure 1d [29] value of 0.74; the 0.81 value reported separately at 60 °C is discussed in Section 7. Binding energies describe modeled complex stability, and conductivity−derived activation parameters describe bulk−transport temperature dependence.

Multiple crosslinking and interpenetrating networks describe network architecture, rather than an exclusive fabrication route. They can accompany casting, support impregnation, or thermal and photoinduced curing. Huang et al. combined covalent junctions with noncovalent interactions in a multiple-crosslinked ionogel and reported mechanical and ion-transport responses for the resulting formulations [29]. Igbaroola et al. used AA/HDDA copolymerization with in situ phase separation to form a self-standing ionogel [23]. Phase separation alone should not be assumed to create a continuous conducting phase. Figure 3 distinguishes reaction schematics (a,c), coordination models with DFT binding energies (b), and measured stress–strain, temperature-dependent conductivity, and polarization responses (d). Binding energies and conductivity-derived apparent activation energies describe different quantities and are not direct measurements of Li^+^ desolvation kinetics. These examples connect molecular coordination and network design, with conclusions limited to the composition and test conditions of each study [22,29].

### 4.6. Organic–Inorganic Hybrids and Thin-Film Engineering

Hybrid formation should distinguish the addition of preformed filler particles from sol–gel reactions that generate an inorganic network. Li et al. used a nonaqueous sol–gel route to immobilize LiTFSI/IL in a pyrrolidinium PIL–silica matrix [39]. Filler chemistry, loading, dispersion, reaction conditions, and shrinkage can affect both mechanical integrity and transport; agglomeration or cracking can offset reinforcement [6,48]. Thin-film integration and photopatterning are separate processing capabilities. Ashby et al. reported spin-coated 600 nm inorganic ionogel films and, separately, UV-enabled photopatterning [49]. These are useful integration comparisons, rather than direct evidence that polymer photocuring yields the same film thickness or battery response. Thickness, electrode coverage, defect tolerance, and through-plane resistance should be assessed for each route and architecture [50,51].

Table 2 summarizes fabrication sequences, selection criteria, representative evidence, and checks. Casting is useful for formulation screening, but drying can dominate preparation time [31]. Scaffold impregnation separates support formation from loading; conductivity and uptake do not replace retention tests [16]. For electrode integration, precursor access and cure location are decisive [36,44]. For the supported IPN-GPE 3/7 membrane, 2 h soaking followed by 12 h curing at 70 °C yielded a total conductivity of 1.30 mS cm^−1^ at 30 °C, a tensile strength of 7.3 MPa, and 82% elongation [44]. This example illustrates how route selection can combine transport, mechanical requirements, and component compatibility. No route is universally preferred: comparable mechanics, IL retention, independent-batch statistics, area uniformity, yield, and total processing time are required before selecting a route for manufacturing.

**Table 2:** Fabrication routes for polymer-based ionogels: processes, selection criteria, evidence, and key checks.

Route ^a^	Key Steps and Structure	Selection Criteria	Representative Evidence ^b^	Key Checks ^g^
Casting/ direct mixing [30,31]	Cast: dissolve → coat → dry. Mix: homogenize without added volatile solvent.	Formulation screening; preformed films.	σ = 3.4 ± 0.3 (*n* = 5) [31]. σ > 1, in-plane [30]. ^c^	Miscibility/viscosity; residual solvent/water; mixing/thickness uniformity; drying demand; IL migration.
Phase inversion/ impregnation [15]	Evaporation-induced phase separation [15] → porous support → impregnation.	Separate support/electrolyte optimization.	PVDF-HFP:PMMA = 40:60 wt%; DMAC/EG [15].	Pore distribution/connectivity; solvent/non-solvent removal; retention under pressure.
Electrospinning/ impregnation [16,17,52,53]	Spin → dry → impregnate (2 h); fibrous scaffold [16].	Adjustable porosity; membrane handling.	PAN/PMMA: 86% porosity; 480% uptake; σ = 3.6 [16]. ^d^	Fiber/thickness uniformity; residual solvent; mat strength; retention under load.
Thermal polymerization [44,54,55]	Precursor infiltration → heat; network formation.	Supported networks; thermally compatible components.	PVDF-HFP: 2 h soaking → 70 °C, 12 h under Ar [44]. ^e^	Conversion; thermal uniformity; shrinkage; residual reactants; component compatibility.
Photoinduced polymerization [24,36]	Shape/coat → irradiate; network formation.	Optically accessible surfaces; electrode integration.	5 min, 250 mW cm^−2^ → 60 °C vacuum, 24 h [24]. separator-free LFP integration [36]. ^e^	Optical access; dose/depth uniformity; conversion; residuals; oxygen inhibition where relevant.
Block copolymer self-assembly [46]	Selective solvation → domain association; physical junctions.	Physical gelation; domain control.	SOS/[BMIM][PF_6_]: gelation at 5 wt% SOS [46]. ^f^	Domain connectivity; directional σ; creep; thermal history; reprocessability.
Hybrid sol–gel [39]	Sol–gel reaction in polymer/IL; inorganic network.	Joint tuning of reinforcement and transport.	LiTFSI/IL in pyrrolidinium PIL–silica; nonaqueous sol–gel [39]. ^f^	Reaction/aging; residuals; dispersion; shrinkage/cracking; brittleness; IL retention.

σ values are in mS cm^−1^ at 25 °C. Examples are formulation-specific, not a process ranking. (a) Routes may be combined. Casting/mixing describe preparation, whereas self-assembly/curing form networks; IPNs and multiple crosslinking may accompany several routes (Section 4.5). Morphology depends on formulation and processing. Direct mixing requires compatible components and manageable viscosity; the studies by Safa et al. and Nykaza et al. are casting examples [30,31]. (b) σ is total ionic conductivity, in mS cm^−1^ at 25 °C. Values are composition-specific; thickness, composition, direction, and measurement conditions must be matched for comparison. In the study by Safa et al., the ± term is not defined as SD or SE, and n = 5 does not establish reproducibility across independent batches [31]. (c) Safa et al. used an electrolyte:PIL mass ratio of 80:20, where the electrolyte was 1 M LiTFSI/[EMIM][TFSI] rather than neat IL; preparation involved 20 min evaporation followed by vacuum drying at 90 °C for 72 h [31]. Nykaza et al. prepared the films by casting, anion exchange, and subsequent electrolyte imbibition, and measured conductivity in-plane [30]. (d) In the PAN/PMMA study, electrolyte uptake is defined as (wet mass − dry mass)/dry mass × 100%, rather than the final IL weight fraction [16]. (e) Huang et al. formed a polymer network within a preformed support; this example does not demonstrate curing inside an assembled cell [44]. Igbaroola et al. prepared an acrylic-acid-based network by irradiation followed by vacuum drying [24]. Separately, Wei et al. formed an ionogel on a wetted LFP cathode by UV curing without an additional separator; separator omission is specific to the reported configuration [36]. Individual processing-step durations do not establish total preparation time or manufacturing throughput. (f) The study of SOS-based ion gels illustrates gelation rather than Li-cell performance [46]. Hybrid formation alone does not establish a continuous conducting phase; sol–gel network formation differs from blending preformed fillers [39]. Inorganic thin films and photopatterning remain contextual examples in Section 4.6, rather than direct evidence for photocuring polymers [49]. (g) Checks are assessment requirements, not properties established for every cited study. Verify uniformity across area, through-plane transport continuity, batch variability, time-dependent mechanics and IL retention at specified temperatures/loads/durations, and manufacturing area, yield, and throughput. Reprocessability and thermal stability require formulation-specific validation. Abbreviations: IL, ionic liquid; PIL, polymeric ionic liquid; IPN, interpenetrating polymer network; UV, ultraviolet; LFP, LiFePO_4_; DMAC, N,N-dimethylacetamide; EG, ethylene glycol; SD, standard deviation; SE, standard error. Polymer abbreviations are defined in Table 3.

**Table 3:** Material-level transport and mechanical properties of representative ionogel electrolytes.

Representative System/Formulation	Processing Route	Total σ (mS cm^−1^; T)	Li-Related Metric/Method	Structure/Mechanical Evidence
PAN/PMMA (4:1 mol) + 1 M LiTFSI/PYR_14_TFSI–PEGDME (2:1 mass) [16]	Electrospun, impregnated	3.60 (25 °C) TP–SS; 10 mV	NR	Porosity 86%; uptake 480% (not mechanical modulus)
PDADMA–TFSI + 1 M LiTFSI/EMIm–TFSI (electrolyte:PIL 80:20 wt) [31]	Cast PIL/IL membrane	3.4 ± 0.3 (25 °C; n = 5)	0.41 (DC/EIS); interface-R limitation	Freestanding; quantitative test not confirmed
PILBCP–TFSI (20 mol% PIL) + 1 M LiTFSI/EMIm–TFSI; imbibed liquid 67 ± 5 wt% [30]	RAFT/cast/imbibe	>1.0 (25 °C) IP–4e; 500 mV	NR	Freestanding film; numerical test unreported
IPN-GPE 3/7: MHBIm–TFSI/PEGDA, EMIm–TFSI, 0.5 M LiTFSI; PVDF–HFP [44]	Electrospun support; in situ IPN	1.30 (30 °C)	NR	Tensile strength 7.3 MPa; elongation 82%
GPE-2: ETPTA/MPC (1:1 molar) + [C_4_mim][TFSI]/LiTFSI [22]	In situ radical cure	0.440 (RT); SS|GPE|SS	0.54 (DC polarization/EIS; 25 °C)	Indentation vs. IL loading; GPE-2 modulus not isolated
IG-0.1PC: BA/EGDMA + Pyr_14_TFSI/LiBF_4_ + PC (9.50 wt% of total precursor, calculated) [33]	In situ thermal cure	1.72 (25 °C) TP–SS; 10 mV	0.41 (DC/EIS)	Reported tensile strength 15 kPa; elongation ~170%; compression stress 14.8 MPa at 90% strain
PAA + 70 wt% 1 m LiFSI/P_111i4_FSI electrolyte [24]	In situ UV cure	≈1.5 (RT)	D(Li) = 9.28 × 10^−11^ m^2^/s (80 °C; PFG)	Long-term mechanical tests not verified
PAA + 70 wt% 3 m LiFSI/P_111i4_FSI electrolyte [24]	In situ UV cure	≈1.0 (RT)	D(Li) = 7.16 × 10^−11^ m^2^/s (80 °C; PFG)	Long-term mechanical tests not verified
AA/HDDA (6.5/6.5 wt%) + 85 wt% 3 M LiFSI/P_111i4_FSI; MBA 1 wt% [23]	UV-cured, phase-separation design	≈2.50 (30 °C) TP–SS; 150 mV	≈0.45 (DC/EIS)	DMA E′ ≈ 2 MPa (30 °C), 1 MPa (50 °C)
IGE-2: VOMImTFSI/PET4A/PEGDA + LiFSI/Pyr_13_FSI [29]	UV multiple crosslinking	0.958 (25 °C) TP–SS; 10 mV	0.74 (Figure 1d [29], author-reported); 0.81 (60 °C, separately reported)	Tensile stress ~0.8 MPa; elongation ~90%

Note: Values are presented as reported in the cited studies, with select within-study sample details verified from the original text or supporting information. Direct comparison requires consideration of measurement temperature, membrane geometry, electrode configuration, excitation amplitude, and test protocol. σ denotes total ionic conductivity rather than Li^+^-only conductivity; a high σ does not itself establish Li^+^-selective transport or through-thickness percolation. Mechanical values are measurement-specific and should not be directly ranked across tensile testing, indentation, and DMA. Measurement and interpretation notes: The conductivity of the PAN/PMMA-supported electrolyte was measured through-plane using stainless-steel (SS) blocking electrodes with a 10 mV excitation amplitude [16]; for the PDADMA–TFSI gel electrolyte, the reported Li^+^ transference number of 0.41 was obtained by DC polarization with impedance-based resistance correction. The authors noted limitations associated with interfacial resistance, and the ± term accompanying the reported conductivity was not identified as SD or SE [31]; the conductivity of the PIL diblock-copolymer electrolyte was measured in-plane using a four-electrode method with a 500 mV AC excitation amplitude [30]; for the ETPTA–MPC system, the main article reports an ETPTA:MPC monomer molar ratio of 1:1, a room-temperature conductivity of 0.440 mS cm^−1^, and a DC-polarization/EIS Li^+^ transference-number estimate of 0.54 at 25 °C. Sample-specific formulations should be distinguished using the preparation information [22]; the conductivity of the AA–HDDA ionogel was measured using a 150 mV AC excitation amplitude. Its reported E′ values are DMA storage moduli and should not be interpreted as tensile strength [23]; for the PAA-supported ionogels, conductivity was reported near room temperature, whereas the Li self-diffusion coefficients were measured by ^7^Li PFG-NMR at 80 °C. These diffusion coefficients are distinct from electrochemical Li^+^ transference numbers [24]; for the PC-containing ionogel, the PC fraction listed in Table 3 is calculated from the reported precursor masses. The main text reports a tensile strength of 15 kPa and an elongation of approximately 170%. Figure 1c is identified as a tensile measurement in its caption, although its vertical axis is labeled “Compressional Stress/kPa”. The compression stress of 14.8 MPa refers to 90% strain in Figure 1d [33]. For IGE-2, the tabulated Li^+^ transference number of 0.74 follows Figure 1d and its accompanying discussion. A value of 0.81 is reported separately at 60 °C, whereas another main-text passage summarizes a room-temperature value of 0.78. These values should remain associated with their respective source locations and measurement conditions [29]. Reporting convention: NR denotes a value not reported for the stated formulation, metric, and measurement context in the reviewed source. It does not mean a value of zero and does not, by itself, establish that a measurement was never performed. An electrochemical Li^+^ transference number is not reported for the PAN/PMMA-supported electrolyte [16]. The corresponding metric is not reported for the PIL diblock-copolymer electrolyte [30]. It is also not reported for the IPN-GPE 3/7 membrane [44]. In the PAA ionogel study, Li self-diffusion coefficients are reported separately, whereas the electrochemical Li^+^ transference number is NR for the tabulated formulations [24]. RT denotes room temperature. A dash, where used, indicates that no comparable value is tabulated; it does not establish the absence of a measurement. Abbreviations: PAN, polyacrylonitrile; PMMA, poly(methyl methacrylate); TFSI, bis(trifluoromethanesulfonyl)imide; LiTFSI, lithium TFSI; PYR_14_TFSI, N-butyl-N-methylpyrrolidinium TFSI; PEGDME, poly(ethylene glycol) dimethyl ether; PDADMA-TFSI, poly(diallyldimethylammonium) TFSI; EMIm-TFSI, 1-ethyl-3-methylimidazolium TFSI; MMA, methyl methacrylate; MUBIm-TFSI, 1-[2-(methacryloyloxy)undecyl]-3-butylimidazolium TFSI; MHBIm-TFSI, 1-[2-(methacryloyloxy)hexyl]-3-butylimidazolium TFSI; PEGDA, poly(ethylene glycol) diacrylate; PVDF-HFP, poly(vinylidene fluoride-co-hexafluoropropylene); ETPTA, ethoxylated trimethylolpropane triacrylate; MPC, 2-methacryloyloxyethyl phosphorylcholine; BA, butyl acrylate; EGDMA, ethylene glycol dimethacrylate; PC, propylene carbonate; PAA, poly(acrylic acid); AA, acrylic acid; HDDA, 1,6-hexanediol diacrylate; MBA, N,N′-methylenebisacrylamide; LiFSI, lithium bis(fluorosulfonyl)imide; PET4A, pentaerythritol tetraacrylate; VOMImTFSI, 1-vinyl-3-ethoxy-methyl-imidazolium TFSI; Pyr_13_FSI, N-propyl-N-methylpyrrolidinium bis(fluorosulfonyl)imide; P_111i4_FSI, trimethyl(isobutyl)phosphonium bis(fluorosulfonyl)imide; [C_4_mim][TFSI], 1-butyl-3-methylimidazolium TFSI; σ, total ionic conductivity; tLi^+^, Li^+^ transference number as reported in each source; DLi, Li self-diffusion coefficient; IL, ionic liquid; PIL, polymeric ionic liquid; PILBCP, poly(ionic liquid) block copolymer; IPN, interpenetrating polymer network; UV, ultraviolet; SS, stainless steel; EIS, electrochemical impedance spectroscopy; DC/EIS, DC polarization corrected by pre/post EIS; PFG-NMR, pulsed-field-gradient nuclear magnetic resonance; DMA, dynamic mechanical analysis; E′, storage modulus measured by dynamic mechanical analysis (not G′ shear storage modulus); RT, room temperature; NR, not reported.

## 5. Structure–Property Relationships

**Figure 4:**
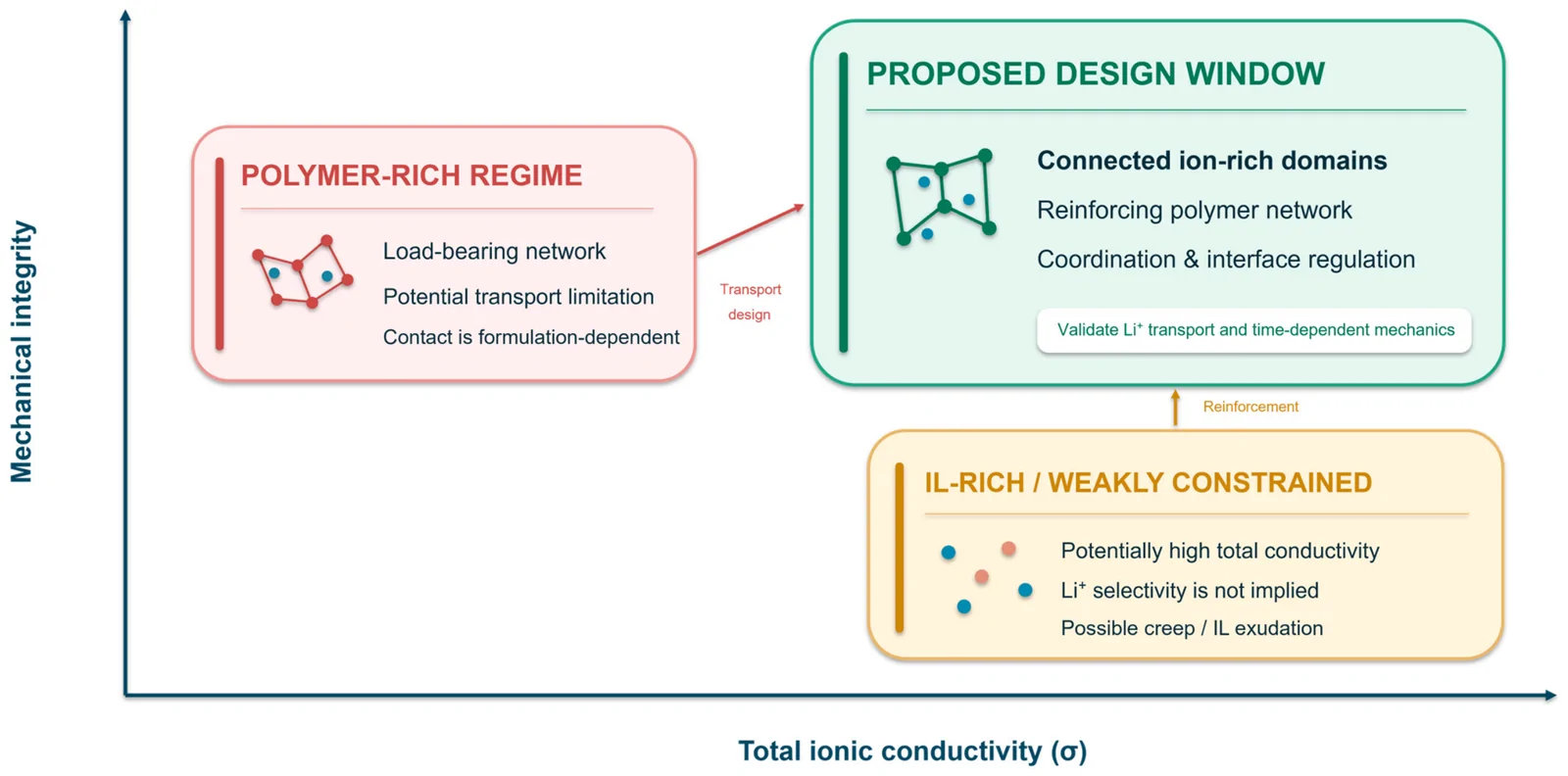
Qualitative structure–property design map for polymer-based ionogels. The polymer-rich regime emphasizes mechanical reinforcement but may constrain ionic transport, whereas the IL-rich, weakly constrained regime may offer higher total ionic conductivity at the expense of mechanical integrity and electrolyte retention. The proposed design window combines connected ion-rich domains, a reinforcing polymer network, and coordination/interfacial regulation to balance transport and mechanical performance. The axes and regime boundaries are conceptual rather than quantitatively determined. Li^+^-specific transport, through-thickness pathway connectivity, and long-term creep resistance require independent experimental verification.

Figure 4 provides a qualitative map of the competing transport and mechanical requirements rather than a quantitative ranking of ionogels. The target is to combine through-plane ionic connectivity with resistance to time-dependent deformation. Section 5.1, Section 5.2, Section 5.3 and Section 5.4 examine the compositional and structural variables governing this balance.

### 5.1. Ionic Liquid Content, Glass-Transition Temperature, and Ionic Conductivity

Increasing IL content often plasticizes the polymer matrix and can increase free volume and the population of mobile ions while lowering the glass-transition temperature (T_g_) and crystallinity. These changes can increase ionic conductivity (σ), but the magnitude and direction of the response depend on ion association, polymer–IL compatibility, and the continuity of the ion-rich phase [4,14,20]. Swelling can reduce the density of elastically active chains per unit volume without necessarily breaking covalent crosslinks [56,57]. Therefore, the optimum composition should not be determined solely from a plot of σ versus IL content (wt%). Instead, the storage modulus (G′), loss modulus (G″), creep compliance, and IL exudation should also be evaluated at the same temperature [58]. Within a multi-crosslinked system, Huang et al. reported total conductivities of 0.244, 0.582, and 0.958 mS cm^−1^ at 25 °C for PE, IGE-1, and IGE-2, respectively, along with a decrease in Tg from −10.6 °C (PE) to −27.3 °C (IGE-2) [29]. In another formulation, imbibition of a PIL diblock copolymer with 67 ± 5 wt% LiTFSI/EMIm-TFSI decreased Tg from 29 to −84 °C and increased the total conductivity to above 1 mS cm^−1^ at 25 °C [30]. Both comparisons involve changes in liquid-electrolyte content and local ionic environment; they should not be used to attribute conductivity improvement exclusively to network topology or preferential Li^+^ conduction.

Vogel–Tammann–Fulcher (VTF) behavior suggests that ion transport is coupled to structural relaxation. However, in phase-separated ionogels, the IL-rich and polymer-rich phases may exhibit distinct relaxation behaviors. A VTF fit is consistent with relaxation-coupled transport but does not uniquely establish its mechanism. In multiphase ionogels, the fitted parameters and any apparent Arrhenius activation energy should be interpreted alongside phase-specific relaxation and connectivity.

### 5.2. Crosslinking Density and Network Topology

Increasing crosslinking density can improve IL retention and elastic recovery, although these gains are often accompanied by smaller network mesh sizes and slower polymer segmental motion. The magnitude of this trade-off depends on network topology, the reversibility of the crosslinks, and the spatial distribution of the ion-rich phase. Networks that combine a mechanically robust skeleton with a compliant ion-rich phase can improve the balance between mechanical integrity and ion transport [23,29,44]. Dynamic reversible bonds can also support network repair and self-healing, as demonstrated in a chemically crosslinked ionogel [59]. Under prolonged direct-current (DC) polarization, however, ionic rearrangement and bond exchange may alter the time-dependent mechanical response of the network; coupled electrochemical and mechanical measurements would be required to test this possibility.

The mechanical metric and test protocol must be identified before comparing network strategies. For example, an AA–HDDA copolymer ionogel containing 85 wt% liquid electrolyte exhibited a DMA storage modulus of approximately 2 MPa at 30 °C and 1 MPa at 50 °C [23]. For the complete PVDF-HFP-supported IPN-GPE 3/7 composite membrane, Huang et al. reported a tensile strength of 7.3 MPa and 82% elongation at break (SI Figure S1) [44]. The IGE-2 multi-crosslinked formulation showed an approximately 0.8 MPa tensile stress and 90% elongation at break [29]. These quantities characterize distinct deformation modes and composite structures, not a standardized ranking of long-term mechanical resistance. None of these short-time metrics by itself establishes creep resistance or quantified IL retention under sustained stack pressure.

Short-time mechanical response must be distinguished from deformation and electrolyte retention under sustained stack pressure. In the AA–HDDA study, the DMA storage modulus decreased from approximately 2 to 1 MPa between 30 and 50 °C, but this frequency-dependent result is not a creep or time-resolved exudation measurement [23]. The authors also proposed that slight IL release under assembly pressure could promote electrode wetting, but the released mass was not quantified [23]. Consequently, sustained-load tests should document time-dependent strain, thickness change, IL mass loss, and recovered conductivity at defined temperature and pressure.

Crosslinking density should therefore not be treated as a single bulk parameter independent of network architecture. In a uniformly crosslinked single network, increasing the number of junctions can simultaneously increase stiffness and restrict segmental relaxation throughout the electrolyte. By contrast, appropriately designed interpenetrating or multiple-network structures can distribute mechanical reinforcement and ion transport across complementary structural elements [23,29,44]. Such functional differentiation does not necessarily require spatial phase separation. The claimed benefit must ultimately be tested at matched electrolyte content and temperature, with separate measurements of directional transport and sustained-load integrity.

### 5.3. Microphase Separation and Continuous Ion-Conduction Pathways

Phase separation can improve the transport–mechanical balance when mechanically reinforcing domains form a sufficiently connected load-bearing network and the ion-rich phase provides a continuous pathway across the electrolyte thickness. Under these conditions, the trade-off between mechanical reinforcement and ion transport can be mitigated, although the two properties remain coupled through phase fraction, interfacial morphology, and transport tortuosity [23,27,28,35]. Through-plane transport can be hindered by isolated IL-rich domains or by lamellar morphologies in which a poorly conducting phase interrupts the through-thickness pathway. Lamellae aligned parallel to the electrode surfaces are not intrinsically blocking; their influence depends on the continuity and conductivity of each phase and should not be inferred from morphology alone [60,61]. Therefore, small-angle X-ray scattering (SAXS) or transmission electron microscopy (TEM) characterization should be presented together with bulk ionic conductivity (σ), and, where possible, direction-dependent conductivity and morphological changes after electrode contact should also be evaluated.

Within-study controls help separate structural effects from cross-publication differences. Using the same LiTFSI/PYR_14_TFSI/PEGDME liquid phase, Rao et al. reported 25 °C total conductivities of 3.6, 2.3, and 1.2 mS cm^−1^ for electrospun PAN/PMMA, electrospun PAN, and Celgard PE supports, respectively, with electrolyte uptakes of 480%, 420%, and 120% [16]. This association supports the importance of porous support architecture and electrolyte uptake, but does not independently isolate tortuosity or Li^+^-specific conduction. In a PIL diblock-copolymer film, Nykaza et al. observed a broad SAXS peak (36.0 nm Bragg spacing) and weakly phase-separated morphology after LiTFSI/EMIm-TFSI imbibition; the reported conductivity exceeding 1 mS cm^−1^ at 25 °C was measured in plane rather than across the membrane thickness [30]. These observations support morphology–total-conductivity associations, not a directly imaged, percolating through-plane Li^+^ pathway.

A more direct within-study comparison was reported by Cheng et al. for homogeneous (H-PE) and phase-separated (PS-PE) PEO/LiTFSI/EmimCl ionogels [62]. SEM and two-dimensional Raman mapping identified interconnected polymer-rich and ion-rich domains in PS-PE. At 30 °C, the Bruce–Vincent Li^+^ transference-number estimate increased from 0.64 for H-PE to 0.89 for PS-PE. The authors also reported a tensile-test modulus of 0.8 MPa and strain exceeding 1700% for PS-PE, compared with 0.5 MPa and approximately 1200% for H-PE. In composite cathodes incorporating these matrices, the effective ionic conductivity increased from 0.53 × 10^−5^ to 1.75 × 10^−5^ S cm^−1^, whereas electronic conductivity remained similar (6.33 × 10^−5^ and 6.41 × 10^−5^ S cm^−1^). The membrane transference and mechanical measurements and the cathode effective conductivity are distinct measurements within the same study, not interchangeable properties of one specimen. Together, they provide experimental support for combining an interconnected ion-rich phase with a mechanically supporting network beyond a bulk-conductivity correlation. Because fabrication solvent and coordination also changed, the improvement cannot be assigned to connectivity alone; the polarization-derived transference number is a method-dependent estimate rather than a direct Li self-diffusion measurement.

To test a topology-controlled transport mechanism directly, comparisons should hold the IL and salt composition, electrolyte uptake, temperature, and membrane thickness as constant as practicable while varying phase connectivity. Direction-dependent EIS, species-resolved transport measurements, and topology-resolved structural characterization must then be evaluated on matched material states. Convergent structural, Li-related transport, and mechanical measurements can support the design principle, while simultaneous changes in coordination, plasticization, or wetting limit attribution to connectivity alone.

Species-resolved diffusion also reveals a concentration-dependent confinement response. At 80 °C, Igbaroola et al. measured Li self-diffusion coefficients of 1.57 × 10^−10^ and 9.28 × 10^−11^ m^2^ s^−1^ in non-confined and confined 1 m LiFSI electrolytes, respectively; for 3 m LiFSI, the corresponding values were 6.55 × 10^−11^ and 7.16 × 10^−11^ m^2^ s^−1^ [24]. Thus, confinement reduced Li self-diffusion at 1 m but modestly increased it at 3 m; these PFG-NMR values are not electrochemical transference numbers or spatial maps of the conduction pathway. Likewise, the AA–HDDA ionogel combining approximately 2.5 mS cm^−1^ total conductivity at 30 °C with temperature-dependent DMA response supports a formulation-level transport–mechanical balance, whereas optical contrast and bending tests do not by themselves resolve the three-dimensional continuity of the ionic phase [23]. A stronger causal claim would require matched-composition controls, directional or through-plane transport, and topology-resolved measurements on comparable material states.

Local coordination offers a complementary explanation that is not equivalent to physical channel continuity. For the zwitterionic GPE-2, the reported room-temperature conductivity of 4.4 × 10^−4^ S cm^−1^ exceeded that of a formulation without MPC (2.4 × 10^−4^ S cm^−1^) [22]. The main article describes an ETPTA:MPC monomer molar ratio of 1:1 and sample series varying the lithium-salt concentration and IL content. Accordingly, differences across these GPEs involve compositional variation as well as coordination. The MPC-free control probes removal of the zwitterionic functionality, but does not by itself separate coordination from the accompanying changes in the precursor and network. In a separate PC-containing ionogel, conductivity increased from 0.47 to 1.72 mS cm^−1^ at 25 °C and the reported transference number increased from 0.10 to 0.41 after PC addition; the polymer-free IL electrolyte also showed a conductivity gain upon PC addition [33]. These controls indicate that liquid-phase solvation and plasticization can contribute even without a designed continuous polymer–IL channel. The causal role of connected ion-rich domains therefore remains best tested by holding liquid composition and uptake constant while changing network topology and measuring directional, species-specific transport.

### 5.4. Performance Comparison of Representative Systems

Table 3 summarizes material-level transport and selected mechanical metrics rather than a universal ranking of fabrication routes. Porous supports, ion-rich compositions, zwitterionic coordination, and multiple-network reinforcement can each alter the reported properties, but their contributions cannot be isolated by comparing maximum σ values across different studies. The relevant assessment is therefore whether the formulation delivers the required transport and mechanical response under a specified measurement geometry and temperature.

Measurement geometry and the definition of the reported transport quantity create additional comparability limits. The PAN/PMMA-supported GPE was measured between stainless-steel blocking electrodes (10 mV; 100 kHz–1 Hz) [16]. The PIL diblock-copolymer electrolyte was measured using an in-plane four-electrode geometry (500 mV; 0.1 Hz–1 MHz) [30]. The AA–HDDA ionogel used blocking electrodes with 150 mV excitation for its total-conductivity measurement [23]. Moreover, Safa et al. cautioned that relatively large interfacial resistance limited the quantitative interpretation of their polarization-derived Li^+^ transference value [31]. Consequently, Table 3 should not be used to rank Li-selective transport or through-plane resistance on the basis of unqualified conductivity maxima; sample identity, temperature, orientation, polarization protocol, and the definition of each transport metric need to accompany the material-level table and any separately reported cell-level comparison.

## 6. Electrode–Electrolyte Interfaces and Cell-Level Validation

**Figure 5:**
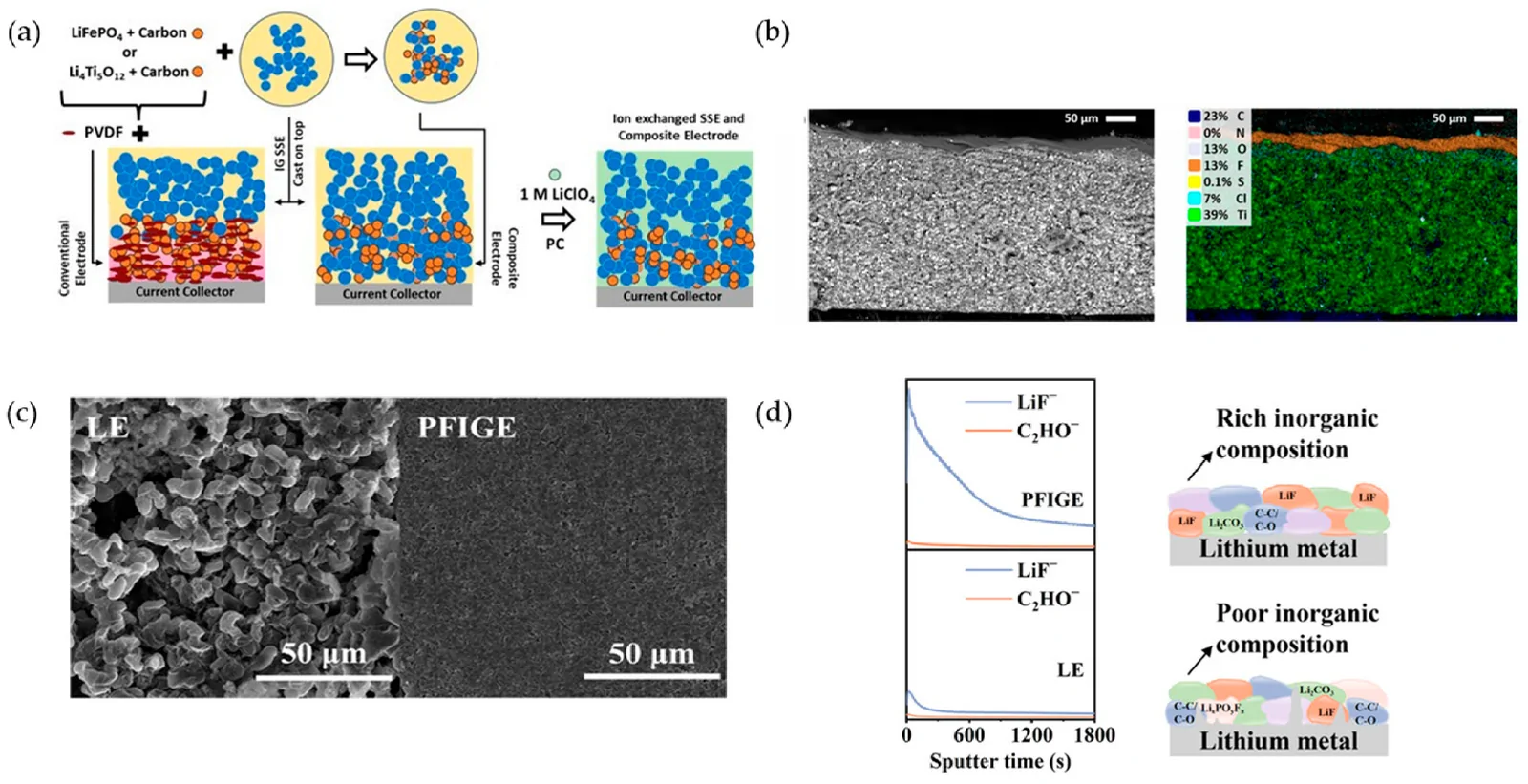
Experimental observations and schematic interpretations of electrode integration and interphase behavior. (**a**) Composite-electrode fabrication and electrolyte-exchange schematic and (**b**) cross-sectional microscopy and elemental mapping, adapted from the cited study; the final exchanged electrolyte contains LiClO_4_/propylene carbonate [63]. (**c**) Post-cycling SEM comparison of lithium surfaces with liquid electrolyte (LE) and polyfluoride ionogel (PFIGE), adapted from Figure 6b of Ref. [64]. (**d**) TOF-SIMS sputter-time profiles of LiF^−^ and C_2_HO^−^ signals and schematic interpretations of SEI composition, adapted from Figure 6e,h [64] of the cited study, respectively; the chemical profiles are measurements, while the SEI arrangement is a proposed interpretation [64]. Both source articles are licensed under CC BY 4.0. The original panels were cropped, rearranged, and relabeled.

Bulk ionic conductivity and mechanical integrity alone do not determine cell performance. Interfacial wetting, Li^+^ desolvation and charge transfer, electrochemical decomposition, current-density distribution, and contact retention collectively reconstruct the electrolyte near the electrodes, producing a SEI at the lithium-metal anode and a cathode–electrolyte interphase (CEI) at the positive electrode. The composition and spatial uniformity of these interphases depend not only on the intrinsic stability of the IL, polymer, and lithium salt, but also on residual monomers, impurities, electrode chemistry, applied pressure, temperature, and current history. Consequently, an ionogel exhibiting high conductivity or a wide apparent electrochemical stability window may still show rapid polarization, nonuniform lithium deposition, increasing interfacial resistance, or premature cell failure. This section therefore connects molecular and process-induced structures to dynamic electrode interfaces and practical cell behavior. Figure 5 illustrates this connection at two complementary scales. Panels (a,b) illustrate electrolyte integration within a composite electrode and the resulting contact topology. Because the reported system involves electrolyte exchange with LiClO_4_/propylene carbonate, these panels are used as a processing comparison rather than as direct evidence for an exclusively IL-based electrolyte phase [63]. Panel (c) presents post-cycling lithium morphology, whereas panel (d) combines TOF-SIMS sputter-time profiles with a schematic interpretation of SEI composition [64]. These elements provide morphological observations, chemical-analysis results, and a proposed structural interpretation, respectively. Neither LiF enrichment nor the schematic alone establishes uniform Li^+^ flux or long-term interphase protection. Section 6.1 examines lithium-metal interfaces and SEI formation, Section 6.2 addresses cathode and high-voltage interfaces, and Section 6.3 discusses the testing conditions required for meaningful cell-level validation. Section 6.4 extends this assessment to cell-scale concentration gradients and spatial variations in current, pressure, and temperature.

### 6.1. Lithium-Metal Interfaces and SEI Formation

The solid-like elasticity of an ionogel alone is insufficient to guarantee suppression of Li dendrite growth [43,65]. Several factors can act simultaneously, including concentration gradients arising from a low Li^+^ transference number, localized current concentration at micropores, reductive decomposition of the polymer and IL, and loss of interfacial contact. Anion-derived inorganic species can contribute to SEI stabilization, but the resulting composition and protective function depend on the electrolyte and cycling conditions. Specific assignments, including LiF and N- or S-containing products, require direct interphase characterization [21,22,29,42].

Direct evidence for changes in lithium-deposition morphology should be distinguished from electrochemical indicators of cycling stability. Post-cycling SEM reveals more compact lithium deposits with the polyfluoride ionogel (PFIGE) than with the liquid-electrolyte control [64]. Complementary in situ optical imaging at 0.5 mA cm^−2^ shows more uniform deposition with PFIGE, whereas protrusions appear in the liquid-electrolyte control within 2 min. These observations support improved deposition morphology within the sampled regions and observation period; they do not establish the absence of dendrites throughout the electrode or over the entire cell lifetime.

Long-duration symmetric-cell cycling provides complementary electrochemical evidence. For example, the IGE-2 electrolyte sustained Li plating/stripping for 5000 h at 60 °C and 0.05 mA cm^−2^, with an areal capacity of 0.1 mAh cm^−2^ per half-cycle [29]. This result supports stable operation under the specified conditions, but the cycling duration alone does not establish dendrite-free deposition or durability at higher current densities and areal capacities.

When evaluating Li|electrolyte|Li symmetric cells, not only the current density but also the areal capacity per cycle, rest time, temperature, applied pressure, Li thickness, and definition of failure should be reported. Because the critical current density (CCD) depends strongly on the duration of each current step and the accumulation of interfacial damage, direct comparison of CCD values obtained using different testing protocols should be avoided [66].

### 6.2. Cathode and High-Voltage Interfaces

Even when the IL itself exhibits high oxidative stability, oxidative decomposition at high-voltage cathode surfaces can be promoted by the polymer matrix, residual monomers, impurities, and conductive carbon additives. High-voltage compatibility should therefore be assessed for the complete ionogel formulation in contact with the intended cathode, rather than inferred from the properties of the neat IL. Curing after precursor infiltration can improve electrode contact and reduce initial interfacial resistance [36]. The benefit of this approach depends on whether curing preserves electrolyte access within the electrode while producing a sufficiently uniform polymer network. Improved contact can reduce initial resistance, but residual reactive components or nonuniform curing may complicate the interpretation of subsequent interfacial degradation. Processing quality and chemical stability should therefore be evaluated together.

High-voltage cycling has been demonstrated for an in situ polymerized QSPE containing Pyr_14_TFSI [42]. CEI chemistry should nevertheless be evaluated separately from improvements in electrode contact. The relevant question is whether the resulting CEI limits further decomposition while permitting sustained Li^+^ transport. The presence of decomposition products alone does not establish this protective function. Surface-chemical observations should therefore be interpreted alongside changes in impedance, coulombic efficiency, and capacity retention during cycling. These responses may also be affected by contact loss or transport limitations, so their evolution should not be attributed exclusively to CEI chemistry. The stability of high-voltage cells should be validated under practically relevant conditions, including the actual cut-off voltage, cathode loading, negative to positive electrode capacity (N/P) ratio, and electrolyte-to-capacity (E/C) ratio.

### 6.3. Practical Cell Conditions

Practical applicability depends on how electrolyte geometry and material inventories support the intended current and capacity [67]. Electrolyte thickness contributes directly to bulk area-specific resistance, ASR_b = L/σ; at σ = 1 mS cm^−1^, 100 and 20 μm layers give 10 and 2 Ω cm^2^, respectively. Casting-blade gap is a useful nominal coating setting for process reproducibility, with the thickness basis used for conductivity or cell-resistance calculations specified separately. Higher cathode loading raises areal capacity and the areal current required at a fixed C-rate, increasing the importance of electrolyte penetration, concentration transport, and contact throughout the electrode. Lithium excess can compensate for irreversible consumption, and additional electrolyte can maintain wetting and ionic access, but both increase inactive mass and volume. Li thickness or N/P should therefore be reported with its capacity basis, and E/C with the included electrolyte components and mass- or volume-based units. Stack pressure can improve contact while also deforming the network or redistributing liquid; pressure-dependent resistance, thickness stability, and liquid retention should be evaluated together. These parameters determine how laboratory cycling translates to cell-level energy, polarization, and durability under the intended temperature and voltage window.

Different validation tests within the same publication should be considered separately. The IGE-2 study reports a 1000-cycle Li||LFP result at 0.5 C, with 79.3% capacity retention, and a distinct high-loading LFP test using 9 mg cm^−2^ at 0.5 C and 60 °C, with 93.5% retention after 100 cycles [29]. Both LFP tests were conducted at 60 °C; however, the 9 mg cm^−2^ loading of the high-loading test should not be assigned to the 1000-cycle test, for which the specific cathode loading was not identified [29]. Likewise, the IPN-GPE 3/7 study reports ionic conductivity at 30 °C but Li||LFP cycling at 40 °C [44]. These examples show why transport measurements and cell-performance metrics require their own test conditions.

Pouch-cell demonstrations represent an important step toward practical implementation [23,38]. However, cell capacity, electrolyte mass, sealing conditions, gas generation, and safety testing should also be reported. The assessment should account for the mass and dimensions of the complete electrolyte assembly, including any supporting scaffold, rather than only the ionogel formulation. Performance should also be examined after storage to determine whether electrolyte redistribution, changes in electrode contact, or package integrity affect subsequent operation. A convincing demonstration therefore requires both reproducible cycling and sufficient reporting to establish how the electrolyte was integrated into the cell. This allows the benefits of polymer engineering to be assessed against the constraints of the intended device.

Storage observations should also be distinguished from mechanical durability under operating conditions. The IPN-GPE 3/7 membrane retained its macroscopic appearance without visible surface cracking after approximately two months in an inert atmosphere [44]. This observation supports storage stability under the reported conditions, but does not quantify creep or ionic-liquid exudation under sustained pressure and elevated temperature. Covalent networks and reinforcing scaffolds provide plausible means of limiting deformation and liquid redistribution; their long-term effectiveness nevertheless requires time-dependent creep or stress-relaxation measurements, thickness monitoring under load, and quantitative analysis of expelled liquid. Any measured mass loss should be distinguished from water or residual-solvent loss before being attributed to ionic-liquid exudation.

Table 4 summarizes the test-specific operating conditions, outcomes, and reporting gaps for the representative cells.

### 6.4. Cell-Scale Transport and Spatial Uniformity

Uniform nominal composition does not ensure uniform ion transport throughout a cell. Variations in electrolyte thickness, local conductivity, curing extent, and electrode infiltration can create spatial differences in resistance before cycling begins. During operation, concentration gradients and changes in electrode contact can introduce additional heterogeneity. These initial manufacturing variations should therefore be distinguished from the concentration and current distributions that develop dynamically under load.

Through-thickness transport requires particular attention because concentration gradients can develop across the electrolyte and within electrolyte-filled porous electrodes. Fawdon et al. directly tracked concentration profiles in a LiFSI/tetraglyme liquid electrolyte using operando Raman microspectroscopy [68]. Their measurements demonstrate how spatially resolved concentration data can reveal transport limitations that a single bulk conductivity value cannot describe. This study provides a methodological example rather than direct evidence for ionogels. Applying the approach to multicomponent ionogels requires formulation-specific spectral calibration and transport analysis.

Across the electrode plane, current-collector resistance and tab geometry can produce potential gradients and redistribute the local reaction current. Drummond et al. developed a pouch-cell model validated against experimental voltage and temperature data and predicted that in-plane electrode grading could homogenize current distribution [69]. The predicted onset of lithium plating shifted from 2.4 C for the uniform-electrode design to 4.3 C for the graded design. These values are model predictions for the studied lithium-ion cell, rather than experimentally established limits for ionogel-based lithium-metal cells. Nevertheless, the study illustrates why uniform electrolyte composition alone cannot guarantee uniform cell utilization.

For ionogels, spatial variations in pressure and temperature may further couple transport to mechanical deformation and contact retention. Nonuniform pressure can alter local contact resistance and, where the network permits deformation or liquid redistribution, change the effective transport geometry over time. Temperature differences can modify conductivity, interfacial kinetics, and mechanical relaxation. These effects should be evaluated under the intended cell conditions rather than inferred from initial tensile properties or room-temperature conductivity. Local SEM images and interphase chemical maps characterize selected regions and do not directly establish whole-cell ionic-flux uniformity, as illustrated by the observations discussed above [64].

A simple resistance estimate illustrates the role of geometry. For a homogeneous electrolyte, the bulk area-specific resistance is ASR_b = L/σ, where L is electrolyte thickness and σ is through-plane ionic conductivity. At σ = 1 mS cm^−1^, thicknesses of 100 and 20 μm correspond to ASR_b values of 10 and 2 Ω cm^2^, respectively. At 3 mA cm^−2^, the corresponding bulk ohmic drops are 30 and 6 mV. These are illustrative calculations that exclude concentration polarization and interfacial losses; reducing mean thickness alone does not establish spatial uniformity. Evaluation should therefore combine thickness mapping, spatially resolved resistance measurements where feasible, temperature mapping, and concentration or current-distribution measurements under defined operating conditions.

## 7. Mechanism-Oriented Characterization of Structure and Ion Transport

Claims regarding polymer–IL interactions should be supported by multiple lines of evidence rather than by a single FTIR peak shift alone. FTIR and Raman spectra provide evidence of coordination environments and anion association, complemented by NMR chemical shifts that reflect changes in local chemical environments [22]. Species-resolved self-diffusion coefficients obtained by pulsed-field gradient NMR (PFG-NMR) provide a distinct transport observable for examining the relevance of these molecular-level interpretations [24]. Quantitative estimates of free and coordinated anion populations require explicit spectral assignments, fitting procedures, and underlying assumptions. When polymer functionality, IL composition, or salt concentration is varied, changes in coordination and association should be evaluated alongside the corresponding diffusion and electrochemical transport responses. These complementary observations, rather than an isolated spectral shift alone, support the proposed interaction–transport relationship.

For example, competitive-coordination ionogels have been evaluated by Raman/FTIR peak assignments, ^7^Li and ^19^F chemical shifts, and static DFT binding-energy calculations [22]. Addition of propylene carbonate to an ionogel changed the Raman-deconvoluted populations of TFSI-associated species as well as the reported transport response [33]. Such observations support changes in local coordination and ion association. Static binding energies should not be reported as directly measured Li^+^ desolvation activation barriers or ligand-exchange rates.

Taylor et al. provide a complementary link between coordination chemistry and Li-specific transport [70]. For [Li(G4)][TFSI], a pCBMA gel with xZI = nZI/(nZI + nLi) = 0.15 increased the PGSE-NMR Li self-diffusion coefficient from 7.7 to 12.5 × 10^−12^ m^2^ s^−1^ relative to the liquid electrolyte. Room-temperature total conductivity was 1.20 mS cm^−1^ in both cases, whereas the DC-polarization/EIS estimate of tLi^+^ increased from 0.19 to 0.37 and the authors’ estimated σLi^+^ = σtLi^+^ increased from 0.23 to 0.44 mS cm^−1^. Thus, unchanged total conductivity can conceal a change in Li-related transport. The larger ^7^Li chemical shift in the corresponding CBMA monomer solution supports altered local coordination, but chemical shifts were not measured in the polymerized gel. The response is chemistry-dependent: pMPC at the same xZI reduced Li self-diffusion to 5.1 × 10^−12^ m^2^ s^−1^ despite an estimated tLi^+^ of 0.41. These complementary measurements support a coordination–transport relationship without equating a higher transference number with faster Li self-diffusion or treating spectroscopy as a direct measurement of ion-pair dissociation kinetics.

Differential scanning calorimetry (DSC) tracks thermal transitions, including T_g_ where detectable, whereas X-ray diffraction (XRD) assesses crystalline order. SAXS probes characteristic structural length scales, and TEM provides local morphological information. Dynamic mechanical analysis (DMA) and rheology characterize the time-dependent viscoelastic response of the polymer network. X-ray photoelectron spectroscopy (XPS) and time-of-flight secondary ion mass spectrometry (TOF-SIMS) can further be used to characterize the SEI and CEI after electrochemical cycling. These measurements should be interpreted together because molecular coordination, phase organization, and mechanical relaxation describe different aspects of the same electrolyte. Sample composition, conditioning history, and measurement temperature should be documented to establish whether the results represent comparable material states. Where changes after cycling are discussed, equivalent handling and analysis conditions are also needed to support meaningful comparisons.

The morphological and mechanical evidence must also be matched to its length and time scale. SAXS/TEM showed a characteristic spacing of 36.0 nm and weak microphase order for an imbibed PIL diblock-copolymer membrane, but did not establish three-dimensional ionic percolation [30]. In an AA–HDDA copolymer ionogel, DMA storage modulus decreased from approximately 2 MPa at 30 °C to 1 MPa at 50 °C [23]. Neither a local structural correlation nor a short-frequency mechanical measurement substitutes for through-plane Li^+^ flux, sustained-load creep, or quantitative liquid exudation measurements.

Structure–transport hypotheses should be tested using controlled compositional or processing variations and complementary electrochemical measurements. Changing one variable at a time, where feasible, can help distinguish the effects of polymer chemistry from those of IL content or fabrication history. The proposed mechanism should account for the measured transport response and its dependence on the relevant structural variables, rather than rely solely on correlations between composition and conductivity. DC-polarization-based Li^+^ transference numbers should be reported with the analysis method, resistance corrections, and applicable assumptions [71]. When complementary measurements yield different interpretations, the discrepancy should be examined in relation to their measurement conditions and assumptions. Such differences can identify the limits of the proposed mechanism and guide further testing.

These measurement distinctions are material for the interpretation of individual studies. The Bruce–Vincent-type DC-polarization values reported for the AA–HDDA ionogel (approximately 0.45) [23] and coordination-engineered GPE-2 (0.54) [22] are method-specific estimates based on current and interfacial-resistance corrections. Safa et al. explicitly identified interfacial-resistance limitations affecting the quantitative comparison of their reported value (0.41) [31]. By contrast, the Li self-diffusion coefficients measured by PFG-NMR under zero applied electric field [24] and the diffusion quantity calculated from Warburg analysis of EIS [33] are different observables and should not be pooled with polarization-derived tLi^+^ values. Where these methods disagree, the difference should be assessed in the context of ionic correlations, concentration, cell geometry, and electrode interfaces rather than averaged into one transport ranking.

For IGE-2, the author-reported Li^+^ transference-number estimate of 0.74 follows Figure 1d of the original article and its accompanying discussion [29] A value of 0.81 is reported separately at 60 °C, while another passage in the same article summarizes a room-temperature value of 0.78. These values are presented with their respective source locations and measurement conditions. They should be interpreted as method-dependent estimates obtained from polarization measurements, with attention to temperature and interfacial-resistance corrections.

In concentrated multicomponent electrolytes, a transference number must be interpreted with its experimental definition and reference frame. Bruce–Vincent–Evans polarization estimates rely on a particular steady-state model and resistance correction [71]. Interfacial evolution may strongly affect the inferred value, as explicitly discussed for a PIL gel electrolyte [31]. PFG-NMR instead measures equilibrium self-diffusion, which includes motion associated with ion pairs or clusters, whereas Warburg-derived diffusion depends on an impedance model and its boundary conditions [24,33]. Accordingly, reports should specify the transport quantity, reference frame where applicable, cell geometry, polarization and temperature conditions, interfacial corrections, and model assumptions. Where feasible, electrophoretic mobility or complementary concentration-dependent measurements can test conclusions inferred from conductivity and self-diffusion alone. In a related Li-salt/IL liquid system (not a polymer ionogel), electrophoretic NMR revealed correlated Li–anion drift and a Li transport estimate distinct from diffusivity-derived ratios [72].

To evaluate the spatial heterogeneity discussed in Section 6.4, bulk measurements should be complemented, where feasible, by operando concentration profiling, spatially resolved impedance measurements, and temperature mapping; the spatial and temporal resolution of each method should be reported.

## 8. Reporting Standards and Future Design Priorities

For polymer ionogels to progress from composition screening to reproducible lithium-based cells, material claims must be linked to processing history and test conditions. Table 5 therefore proposes a minimum reporting set that allows conductivity, mechanics, and cell behavior to be interpreted as a connected system rather than as isolated performance values.

The proposed structure–property relationships remain conditional because polymer chemistry, liquid content, salt concentration, network structure, and processing history often change together. Consequently, an increase in conductivity or mechanical strength cannot necessarily be assigned to a single structural feature. For example, measurements at 80 °C indicate lower Li self-diffusion upon confinement at the lower nominal salt concentration, but a modest increase at the higher nominal salt concentration [24]. This concentration-dependent response limits a general claim that confinement always improves Li transport; the magnitude of small differences must also be considered alongside measurement uncertainty.

Differences in measured quantities and observation scales impose further limitations. PFG-NMR self-diffusion, polarization-derived transference values, and total ionic conductivity describe different aspects of transport and should not be treated as interchangeable measures. Likewise, initial mechanical properties, storage observations, local lithium morphology, and long-duration cycling probe different material states and timescales. The storage observation does not independently establish resistance to creep or quantitative IL retention under operating conditions [44]. Likewise, the local morphological and chemical analyses do not independently establish whole-cell transport uniformity [64].

At the molecular level, future designs should prioritize coordination environments that combine sufficient salt dissociation with rapid ligand exchange [74]. Maximizing polymer–Li^+^ binding strength alone can immobilize Li^+^ and increase desolvation resistance. Functional groups, fixed charges, and zwitterionic motifs should therefore be evaluated by their effects on both total conductivity and the effective Li^+^ contribution [74,75].

At the network level, the central question is whether pathway connectivity can be modified independently of liquid content and polymer chemistry to improve Li transport without compromising mechanical durability. Controlled comparisons should maintain composition, conditioning, measurement temperature, and specimen geometry as closely as feasible while varying the processing conditions that determine morphology. Structural characterization should then be combined with Li self-diffusion, polarization-derived transport measurements, and time-dependent mechanical testing on corresponding formulations. If improved connectivity increases only total conductivity without improving Li-specific transport, the proposed Li-transport benefit would require qualification. Similarly, if the morphology or resistance changes substantially under sustained pressure, an initially favorable network cannot be assumed to remain effective during operation. At the manufacturing and cell levels, scale-up claims require evidence of uniform curing or impregnation over practical areas, controlled residual solvent or monomer, low area-specific resistance, and stable behavior during storage and pressure changes. The review’s framework should therefore be used as a design hierarchy—interaction, microstructure, transport, mechanics, and interface—rather than as a universal ranking of materials tested under dissimilar conditions.

Practical competitiveness should be evaluated against a conventional liquid-electrolyte control and, where relevant, an established solid-electrolyte system selected for the same target application. Comparisons should use matched electrode chemistry, cathode loading, lithium inventory, voltage window, temperature, and cycling conditions as closely as feasible [67]. Architecture-dependent differences, including electrolyte thickness, supporting layers, added liquid, and required stack pressure, should be reported and included in the assessment of cell mass, volume, polarization, and durability. Capacity retention should be accompanied by the delivered areal capacity, current density, cycle count, and stated failure criterion.

Table 6 organizes application-matched benchmarking into five assessment areas: cycling and delivered capacity, energy and material inventories, contact and mechanical retention, cell safety, and manufacturing and reproducibility. N/P and E/C definitions should be consistent across the compared cells Cycling should be evaluated using initial and retained areal capacity, polarization, and cycle count at a common retention endpoint, with the failure mode stated. Energy comparisons should include the mass and volume of the complete electrolyte assembly, and the required operating pressure should be reported. Mechanical retention, cell-level abuse outcomes under comparable test conditions, and independent-batch processing data should be evaluated alongside cycling performance. Manufacturing comparisons should account for processed area, total preparation time, uniformity, yield, variability, and residuals.

## 9. Conclusions

Polymer-based ionogels are best understood as process-defined electrolytes rather than simple mixtures of a polymer and an IL. Polymer–ion interactions shape the structures formed during fabrication, while phase connectivity, network relaxation, and interfacial reactions together determine whether favorable bulk properties translate into stable cell operation.

The central objective is to combine continuous ion-transport pathways with a thin, mechanically durable framework while improving the Li^+^ contribution to transport. Competitive coordination, fixed charges, multiple-crosslinking strategies, phase separation, and in situ curing provide promising design approaches, but their benefits depend on composition, processing history, and operating conditions. Progress should be judged by matched-condition evidence linking structure to Li-specific transport, time-dependent mechanical integrity, interphase evolution, and cell performance. High bulk conductivity, initial tensile strength, or prolonged low-current cycling alone is insufficient to establish practical competitiveness. The remaining questions concern whether favorable pathways persist under pressure and cycling, whether IL retention accompanies long-term mechanical stability, and whether local interfacial improvements translate into uniform cell operation. Application-specific comparisons with liquid and established solid electrolytes should therefore account for electrode loading, lithium and electrolyte inventories, pressure, temperature, voltage, durability, and the complete electrolyte assembly.

## Figures and Tables

**Table 1:** Interactions among polymers, ionic liquids, and lithium salts and their effects on electrolyte design.

Interaction	Representative Combination	Expected Effect	Potential Issue When Excessive
Coulombic/Electrostatic Interaction [18,19,20]	Fixed charges in PILs + IL counterions [18,19]	Enhanced IL affinity; architecture-dependent ion mobility [18,19,20]	Increased T_g_ and reduced ionic mobility [19,20]
Ion–Dipole Coordination [14,21,22]	Li^+^–EO, Li^+^–C=O, Li^+^–N≡C [14,21,22]	Salt dissociation and Li^+^ solvation [21,22]	Strong Li^+^ binding and restricted mobility [14,21]
Hydrogen Bonding [13,23,24,25,26]	Imidazolium C–H···O, COOH···anion [23,24,25]	Improved miscibility, reversible crosslinking, and interfacial adhesion [13,23,24]	Highly sensitive to anions and moisture [25,26]
Solvophobic Interaction/Microphase Separation [12,23,27,28]	IL-philic/IL-phobic blocks [12,27,28]	Separation of mechanically reinforcing and ion-conducting domains [23,27,28]	Discontinuous domains can reduce ionic conductivity (σ) [27,28]
Cation–π/directional polymer–IL interactions [13]	Aromatic polymers or PILs + IL cations [13]	Enhanced polymer–IL compatibility and stabilization of mixed phases [13]	Temperature-dependent weakening of directional interactions can induce phase separation [13]
Multiple Interactions [29]	Covalent network + H-bonding + polycation–anion–Li^+^ coordination [29]	Enhanced mechanical robustness while maintaining/promoting Li^+^ transport [29]	Excessive crosslink density may suppress segmental mobility and reduce ionic transport [29]

Abbreviations: PILs, poly(ionic liquid)s; IL, ionic liquid; T_g_, glass-transition temperature; EO, ethylene oxide.

**Table 4:** Test-specific operating conditions and outcomes of representative ionogel-based cells.

Parameter	Li||Li Symmetric-Cell Test [29]	Li||LFP, 1000-Cycle Test [29]	Li||LFP, High-Loading Test [29]	100-Cycle Test [44]
Electrolyte	IGE-2	IGE-2	IGE-2	IPN-GPE 3/7
Cell configuration	Li||Li symmetric cell	Li||LFP coin cell	Li||LFP coin cell with a high-loading cathode	Li||LFP coin cell
Operating temperature (°C)	60	60	60	40
Current density or C-rate	0.05 mA cm^−2^	0.5 C	0.5 C	0.1 C
Symmetric-cell areal capacity (mAh cm^−2^ per half-cycle)	0.1	N/A	N/A	N/A
Cathode mass loading (mg cm^−2^)	N/A	Not assigned to this test ^a^	9 ^b^	NR
Cycling voltage range (V)	N/A	2.5–3.8	2.5–3.8	2.5–4.0
Li-metal thickness (μm)	NR ^c^	450 ^c^	450 ^c^	NR
Cycling performance	5000 h of plating/stripping	79.3% capacity retention after 1000 cycles	93.5% capacity retention after 100 cycles	94% capacity retention after 100 cycles
Source location in the cited study	Main-text Figure 3d; SI pp. S5–S6 [29]	Main-text Figure 5d; SI pp. S3 and S5 [29]	Main-text discussion of Figure S22; SI pp. S3, S5, and S29 [29]	Main-text Section 2.3 and Figure 5A,B [29]

Notes: Each column represents a distinct cell test. Conditions from different tests within the same publication are not combined. NR, not reported for the specified parameter or test in the source material examined; N/A, not applicable. NR does not indicate a zero value. The cycling voltage range is not applicable to the galvanostatic Li||Li plating/stripping test. Thickness descriptors should state their reporting basis: nominal casting-blade setting, measured membrane thickness, or assembled electrolyte-layer thickness. A casting setting supports process reproducibility, while the thickness used in L/σ calculations should correspond to the conductivity or cell test. Stack pressure, N/P, and E/C should be paired with the specific cell result and their definitions. NR denotes a test-specific value not reported in the source material examined; it is not a zero value. ^a^ The general cathode-preparation procedure reports active-material loadings of approximately 2.5 or 5 mg cm^−2^, but does not identify which loading applies to the 1000-cycle LFP test [29]. ^b^ The 9 mg cm^−2^ loading and 93.5% retention after 100 cycles at 0.5 C and 60 °C follow the main-text discussion of the high-loading LFP test [29]. This loading is assigned only to that experiment. ^c^ A Li-metal thickness of 450 μm is specified in the general LFP/NCM coin-cell assembly procedure [29]. The lithium thickness is not separately specified for the symmetric-cell experiment. Abbreviations: LFP, lithium iron phosphate; IPN-GPE, interpenetrating polymer network gel polymer electrolyte; N/P, negative-to-positive electrode capacity ratio; E/C, electrolyte-to-capacity ratio; SI, supporting information. Source locations refer to the cited original studies.

**Table 5:** Suggested minimum reporting parameters and validation criteria for polymer–ionic liquid ionogel electrolytes.

Category	Essential Reporting Items	Key Checks
Composition [2,3,15,21]	Polymer, IL, lithium salt, and additive contents; concentration bases; purity; water content; residual solvent where relevant [2,3,15,21]	Complete component accounting; consistent concentration definitions; identification of compositional differences between compared samples [3,21]
Fabrication [5,36,44,49]	Precursor composition; initiator and crosslinker; drying and curing conditions; atmosphere; conversion or gel fraction where applicable; processed area [5,36,44,49]	Residual reactive species; curing or impregnation uniformity; batch variation; distinction between curing time and total processing time
Geometry [16,17,23,49]	Mean thickness and variation; test area; porosity; electrolyte uptake; supporting-layer thickness and mass [16,17,23,49]	Through-plane transport geometry; thickness uniformity; inclusion of the complete electrolyte assembly
Ion Transport [3,16,21,24,29,31,33,71]	Conductivity and temperature; measurement direction and cell geometry; electrode type; EIS protocol; definition and method of Li-related transport metrics [3,16,21,29,33]	Measurement uncertainty; comparable material states; distinction between total conductivity, self-diffusion, and polarization-derived transference values [24,31,71]
Mechanical Properties/Long-term Durability [4,23,28,29]	Test mode; temperature; load or pressure; duration; creep or relaxation; thickness changes; amount and identity of expelled liquid where assessed	Separation of initial strength from operating durability; retention of geometry and transport properties; distinction between IL loss and water or solvent loss
Safety [38,39,44,73]	Thermal-analysis conditions; ignition or combustion protocol; cell-level abuse conditions and outcomes; gas analysis where assessed	Direct support for the specific safety claim; distinction between thermal stability, resistance to ignition, and cell-level safety [73]
Interface/Cell [21,23,29,36,38,42,64]	Electrode chemistry; cathode loading and areal capacity; Li thickness or N/P ratio with calculation basis; total electrolyte amount and E/C definition; pressure; temperature; voltage window; current and capacity per step [21,23,29,36,38,42]	Test-specific pairing of conditions and outcomes; evolution of polarization and interfacial resistance; defined failure criteria; separation of morphological evidence from cycling indicators [29,64]
Spatial uniformity [68,69]	Locations and resolution of thickness, concentration, resistance, current, and temperature measurements; operating state; pressure distribution where measured [68,69]	Distinction between local and whole-cell observations; separation of measured distributions from model predictions; changes during cycling [68,69]
Practical benchmarking [67]	Target application; liquid- or solid-electrolyte comparator; matched test conditions; electrolyte assembly mass and volume; delivered capacity, energy, and durability where available [67]	Explicit comparison conditions; inclusion of architecture-dependent requirements; evaluation against application-matched reference cells [67]
Reproducibility	Numbers of independent samples, cells, and batches; uncertainty; exclusions and failures; retention and failure definitions	Consistency across replicates and batches; transparent reporting of variability and unsuccessful tests

Abbreviations: IL, ionic liquid; σ, ionic conductivity; t_Li_^+^, Li^+^ transference number; DMA, dynamic mechanical analysis; TGA, thermogravimetric analysis; DSC, differential scanning calorimetry; E/C, electrolyte-to-capacity ratio; N/P, negative-to-positive electrode capacity ratio; EIS, electrochemical impedance spectroscopy; ASR, area-specific resistance. The reporting criteria are proposed by the authors. Experimental and modelling examples motivate spatially resolved assessment [68,69]. Cell-level benchmarking principles are provided by Randau et al. [67].

**Table 6:** Proposed criteria for application-matched benchmarking of polymer-based ionogel cells against conventional liquid-electrolyte and established solid-electrolyte cells.

Metric	Common Comparison Basis	Criterion or Interpretation
Cycling and delivered capacity [29,66,67]	Matched electrodes, voltage, current, temperature, N/P, and E/C definitions [67]	Compare initial and retained areal capacity, polarization, and cycles to a common retention endpoint. State the failure mode [29,66,67]
Energy and material inventories [67]	Complete electrolyte assembly for each architecture [67]	Include ionogel/support, liquid/separator, or solid-layer/auxiliary-component mass and volume in cell-level comparisons [67]
Contact and mechanical retention [23,28,66]	Intended pressure, temperature, and duration; documented pressure differences [23]	Quantify creep or thickness change, contact resistance, liquid loss, and retained transport performance [23,28,66]
Cell safety [64,73]	Common abuse protocol; comparable cell capacity and state of charge [64]	Compare cell responses and identify the safety claim supported by each test [64,73]
Manufacturing and reproducibility [24,31,49]	Processed area, total preparation time, independent batches, and cell numbers [24,31,49]	Compare uniformity, yield, variability, and residuals. Report total processing time [24,31,49]

Note: These criteria organize an application-matched assessment. Retention endpoints and failure criteria should be defined consistently across the compared cells.

## Data Availability

This review is based on published literature. The data discussed are available in the cited publications and their supporting information. No new experimental data were generated in this study.

## Nomenclature

The symbols and quantities used in this review are defined below, together with their units.

**Symbol or Quantity**	**Meaning**	**Unit or Interpretation**
σ	Total ionic conductivity	S cm^−1^ or mS cm^−1^
t_Li_^+^	Reported Li^+^ transference number	Dimensionless; method and reference frame must be stated
G′, G″	Storage and loss shear moduli	Pa; temperature and frequency dependent
T, T_g_	Absolute temperature; glass-transition temperature	K for inverse-temperature plots; Tg in °C or K as stated
E_a_	Apparent activation energy from a specified fit	eV or kJ mol^−1^; not a directly measured desolvation barrier
E_b_^Li^	Calculated Li^+^ binding energy in Figure 3b	eV; model- and definition-dependent
I, t	Current; elapsed time in Figure 3d	μA; s; t is distinct from tLi^+^
Z′, Z″	Real and imaginary impedance components	Ω
M, m	Molarity; molality	mol L^−1^ of solution; mol kg^−1^ of the specified solvent basis
C-rate	Charge/discharge rate relative to rated capacity	h^−1^; 1 C corresponds to a nominal 1 h charge/discharge
CE	Coulombic efficiency	%; specify charge/discharge convention
ASR	Area-specific resistance	Ω cm^2^; state bulk/interface definition
CCD	Critical current density	mA cm^−2^; protocol dependent
E/C	Electrolyte-to-capacity ratio	e.g., μL mAh^−1^ or g Ah^−1^; specify volume or mass basis
N/P	Negative-to-positive electrode capacity ratio	Dimensionless; state capacity basis
D_Li_	Li^+^ self-diffusion coefficient measured by ^7^Li PFG-NMR	m^2^ s^−1^; Table 3 values at 80 °C; not tLi^+^
E′	Storage modulus from DMA (distinct from shear storage modulus G′)	Pa or MPa; temperature and frequency dependent

## References

[1] Fenton D.E., Parker J.M., Wright P.V. (1973). Complexes of alkali metal ions with poly(ethylene oxide). Polymer.

[2] Armand M., Endres F., MacFarlane D.R., Ohno H., Scrosati B. (2009). Ionic-liquid materials for the electrochemical challenges of the future. Nat. Mater..

[3] Lewandowski A., Świderska-Mocek A. (2009). Ionic liquids as electrolytes for Li-ion batteries—An overview of electrochemical studies. J. Power Sources.

[4] Watanabe M., Thomas M.L., Zhang S., Ueno K., Yasuda T., Dokko K. (2017). Application of Ionic Liquids to Energy Storage and Conversion Materials and Devices. Chem. Rev..

[5] Susan M.A.B.H., Kaneko T., Noda A., Watanabe M. (2005). Ion Gels Prepared by in Situ Radical Polymerization of Vinyl Monomers in an Ionic Liquid and Their Characterization as Polymer Electrolytes. J. Am. Chem. Soc..

[6] Néouze M.-A., Le Bideau J., Gaveau P., Bellayer S., Vioux A. (2006). Ionogels, New Materials Arising from the Confinement of Ionic Liquids within Silica-Derived Networks. Chem. Mater..

[7] Le Bideau J., Viau L., Vioux A. (2011). Ionogels, ionic liquid based hybrid materials. Chem. Soc. Rev..

[8] Chen N., Zhang H., Li L., Chen R., Guo S. (2018). Ionogel Electrolytes for High-Performance Lithium Batteries: A Review. Adv. Energy Mater..

[9] Tripathi A.K. (2021). Ionic liquid–based solid electrolytes (ionogels) for application in rechargeable lithium battery. Mater. Today Energy.

[10] Ma X., Yu J., Hu Y., Texter J., Yan F. (2023). Ionic liquid/poly(ionic liquid)-based electrolytes for lithium batteries. Ind. Chem. Mater..

[11] Zhao S., Shui T., Wang W., Zhang H., Li C., Guo C., Zhang W., Sun Z. (2026). A unified framework to tailor multifunctional ionogel electrolytes for flexible energy storage. Matter.

[12] Ueki T., Watanabe M. (2012). Polymers in Ionic Liquids: Dawn of Neoteric Solvents and Innovative Materials. Bull. Chem. Soc. Jpn..

[13] Watanabe M. (2026). Polymers in ionic liquids and ionic liquids in polymers: Critical factors for compatibility and materialization. Green Chem..

[14] Kumar Y., Hashmi S.A., Pandey G.P. (2011). Lithium ion transport and ion–polymer interaction in PEO based polymer electrolyte plasticized with ionic liquid. Solid State Ion..

[15] Zhai W., Zhu H.-J., Wang L., Liu X.-M., Yang H. (2014). Study of PVDF-HFP/PMMA blended micro-porous gel polymer electrolyte incorporating ionic liquid [BMIM]BF4 for Lithium ion batteries. Electrochim. Acta.

[16] Rao M., Geng X., Liao Y., Hu S., Li W. (2012). Preparation and performance of gel polymer electrolyte based on electrospun polymer membrane and ionic liquid for lithium ion battery. J. Membr. Sci..

[17] Cheruvally G., Kim J.-K., Choi J.-W., Ahn J.-H., Shin Y.-J., Manuel J., Raghavan P., Kim K.-W., Ahn H.-J., Choi D.S. (2007). Electrospun polymer membrane activated with room temperature ionic liquid: Novel polymer electrolytes for lithium batteries. J. Power Sources.

[18] Mecerreyes D. (2011). Polymeric ionic liquids: Broadening the properties and applications of polyelectrolytes. Prog. Polym. Sci..

[19] Yuan J., Mecerreyes D., Antonietti M. (2013). Poly(ionic liquid)s: An update. Prog. Polym. Sci..

[20] Shaplov A.S., Marcilla R., Mecerreyes D. (2015). Recent Advances in Innovative Polymer Electrolytes based on Poly(ionic liquid)s. Electrochim. Acta.

[21] Tu H., Peng K., Xue J., Xu J., Zhao J., Guo Y., Lu S., Wang Z., Chen L., Li H. (2024). Solvation and interfacial chemistry in ionic liquid based electrolytes toward rechargeable lithium-metal batteries. J. Mater. Chem. A.

[22] Li J., Zhang T., Hui X., Zhu R., Sun Q., Li X., Yin L. (2023). Competitive Li+ Coordination in Ionogel Electrolytes for Enhanced Li-Ion Transport Kinetics. Adv. Sci..

[23] Igbaroola B., Demarthe N., Howlett P., Forsyth M., Kvasha G., Kvasha A., Sananes-Israel S., De Meatza I., Dupré N., Le Bideau J. (2025). In-situ phase separated ionogel electrolytes with enhanced properties for lithium metal batteries. Chem. Eng. Sci..

[24] Igbaroola B., Eddahani Y., Howlett P., Forsyth M., O’Dell L., Dupré N., Le Bideau J. (2025). Lithium Diffusion-Efficient Ionogels as Polymer Solid Electrolyte for Next-Gen Lithium-Ion Batteries. Energy Environ. Mater..

[25] Gao Y., Zhang L., Wang Y., Li H. (2010). Probing Electron Density of H-Bonding between Cation−Anion of Imidazolium-Based Ionic Liquids with Different Anions by Vibrational Spectroscopy. J. Phys. Chem. B.

[26] Cammarata L., Kazarian S.G., Salter P.A., Welton T. (2001). Molecular states of water in room temperature ionic liquids. Phys. Chem. Chem. Phys..

[27] Choi J.-H., Ye Y., Elabd Y.A., Winey K.I. (2013). Network Structure and Strong Microphase Separation for High Ion Conductivity in Polymerized Ionic Liquid Block Copolymers. Macromolecules.

[28] Mizuno H., Hashimoto K., Tamate R., Kokubo H., Ueno K., Li X., Watanabe M. (2020). Microphase-separated structures of ion gels consisting of ABA-type block copolymers and an ionic liquid: A key to escape from the trade-off between mechanical and transport properties. Polymer.

[29] Huang J., Hu Y., Gao Q., She Y., Li J., Hu H., Zheng H., Yan F., Ji X. (2025). Multiple-crosslinking-reinforced ionogel electrolytes for safe and high-performance quasi-solid-state lithium metal batteries. J. Mater. Chem. A.

[30] Nykaza J.R., Savage A.M., Pan Q., Wang S., Beyer F.L., Tang M.H., Li C.Y., Elabd Y.A. (2016). Polymerized ionic liquid diblock copolymer as solid-state electrolyte and separator in lithium-ion battery. Polymer.

[31] Safa M., Chamaani A., Chawla N., El-Zahab B. (2016). Polymeric Ionic Liquid Gel Electrolyte for Room Temperature Lithium Battery Applications. Electrochim. Acta.

[32] Frömling T., Kunze M., Schönhoff M., Sundermeyer J., Roling B. (2008). Enhanced Lithium Transference Numbers in Ionic Liquid Electrolytes. J. Phys. Chem. B.

[33] Zhou J., Jiang S., Wang Z., Wang C., Tan S., Wu Y. (2024). Propylene carbonate activated electrochemical performances of in situ polymerized ionogel electrolytes. J. Power Sources.

[34] Porcarelli L., Shaplov A.S., Salsamendi M., Nair J.R., Vygodskii Y.S., Mecerreyes D., Gerbaldi C. (2016). Single-Ion Block Copoly(ionic liquid)s as Electrolytes for All-Solid State Lithium Batteries. ACS Appl. Mater. Interfaces.

[35] Weber R.L., Ye Y., Schmitt A.L., Banik S.M., Elabd Y.A., Mahanthappa M.K. (2011). Effect of Nanoscale Morphology on the Conductivity of Polymerized Ionic Liquid Block Copolymers. Macromolecules.

[36] Wei Z., Zhu S., Zhu X., Yu L., Jiang Z., Tang X., Qu X., Wang Y., Tang H., Liu X. (2024). In Situ-Polymerized Separator-Free Ionogel Electrolyte with Enhanced Interfacial Compatibility for Integrated Solid-State Lithium Batteries. Energy Fuels.

[37] Cheng Y., Zhang L., Xu S., Zhang H., Ren B., Li T., Zhang S. (2018). Ionic liquid functionalized electrospun gel polymer electrolyte for use in a high-performance lithium metal battery. J. Mater. Chem. A.

[38] Que M., Tong Y., Wei G., Yuan K., Wei J., Jiang Y., Zhu H., Chen Y. (2016). Safe and flexible ion gel based composite electrolyte for lithium batteries. J. Mater. Chem. A.

[39] Li X., Li S., Zhang Z., Huang J., Yang L., Hirano S.-I. (2016). High-performance polymeric ionic liquid–silica hybrid ionogel electrolytes for lithium metal batteries. J. Mater. Chem. A.

[40] Sun Y., Gao L., Xie H., Liu J. (2025). Self-healing poly(ionic liquid) gel polymer electrolytes for high-performance lithium metal batteries enabled by ion-dipole interactions. J. Power Sources.

[41] McGrath L.M., Rohan J.F. (2020). Pyrrolidinium Containing Ionic Liquid Electrolytes for Li-Based Batteries. Molecules.

[42] Ma S., Zhang D., Tang Z., Li W., Zhang Y., Zhang Y., Ji K., Chen M. (2024). In Situ Polymerized Quasi-Solid Electrolytes Compounded with Ionic Liquid Empowering Long-Life Cycling of 4.45 V Lithium–Metal Battery. ACS Appl. Mater. Interfaces.

[43] Swiderska-Mocek A., Gabryelczyk A. (2023). Interfacial Stabilizing Effect of Lithium Borates and Pyrrolidinium Ionic Liquid in Gel Polymer Electrolytes for Lithium-Metal Batteries. J. Phys. Chem. C.

[44] Huang B., Zhang Y., Que M., Xiao Y., Jiang Y., Yuan K., Chen Y. (2017). A facile in situ approach to ion gel based polymer electrolytes for flexible lithium batteries. RSC Adv..

[45] Zhang Y., Noè C., Elia G.A., Gerbaldi C. (2024). Ionogel hybrid polymer electrolytes encompassing room-temperature ionic liquids for 4V-class Li-metal batteries operating at ambient temperature. Green Chem. Lett. Rev..

[46] He Y., Boswell P.G., Bühlmann P., Lodge T.P. (2007). Ion Gels by Self-Assembly of a Triblock Copolymer in an Ionic Liquid. J. Phys. Chem. B.

[47] Lodge T.P., Ueki T. (2016). Mechanically Tunable, Readily Processable Ion Gels by Self-Assembly of Block Copolymers in Ionic Liquids. Acc. Chem. Res..

[48] Yang G., Song Y., Wang Q., Zhang L., Deng L. (2020). Review of ionic liquids containing, polymer/inorganic hybrid electrolytes for lithium metal batteries. Mater. Des..

[49] Ashby D.S., DeBlock R.H., Lai C.-H., Choi C.S., Dunn B.S. (2017). Patternable, Solution-Processed Ionogels for Thin-Film Lithium-Ion Electrolytes. Joule.

[50] Hu Y., Ruud A., Miikkulainen V., Norby T., Nilsen O., Fjellvåg H. (2016). Electrical characterization of amorphous LiAlO2 thin films deposited by atomic layer deposition. RSC Adv..

[51] Jetybayeva A., Uzakbaiuly B., Mukanova A., Myung S.-T., Bakenov Z. (2021). Recent advancements in solid electrolytes integrated into all-solid-state 2D and 3D lithium-ion microbatteries. J. Mater. Chem. A.

[52] Xue J., Wu T., Dai Y., Xia Y. (2019). Electrospinning and Electrospun Nanofibers: Methods, Materials, and Applications. Chem. Rev..

[53] Fadil F., Affandi N.D.N., Misnon M.I., Bonnia N.N., Harun A.M., Alam M.K. (2021). Review on Electrospun Nanofiber-Applied Products. Polymers.

[54] Vijayakumar V., Anothumakkool B., Kurungot S., Winter M., Nair J.R. (2021). In situ polymerization process: An essential design tool for lithium polymer batteries. Energy Environ. Sci..

[55] Chen Y., Huo F., Chen S., Cai W., Zhang S. (2021). In-Built Quasi-Solid-State Poly-Ether Electrolytes Enabling Stable Cycling of High-Voltage and Wide-Temperature Li Metal Batteries. Adv. Funct. Mater..

[56] Ghorai A., Alex A., Balmuchu S.P., Banerjee S., Choudhury S. (2025). Polymer-based ionic liquids in lithium batteries. Curr. Opin. Electrochem..

[57] Tamate R. (2025). Development of functional polymer gel electrolytes and their application in next-generation lithium secondary batteries. Polym. J..

[58] Zhang S., Lee K.H., Frisbie C.D., Lodge T.P. (2011). Ionic Conductivity, Capacitance, and Viscoelastic Properties of Block Copolymer-Based Ion Gels. Macromolecules.

[59] Wan L., Cao X., Xue X., Tong Y., Ci S., Huang H., Zhou D. (2022). Self-Healing and Flexible Ionic Gel Polymer Electrolyte Based on Reversible Bond for High-Performance Lithium Metal Batteries. Energy Technol..

[60] Gwee L., Choi J.-H., Winey K.I., Elabd Y.A. (2010). Block copolymer/ionic liquid films: The effect of ionic liquid composition on morphology and ion conduction. Polymer.

[61] Kim S.Y., Yoon E., Joo T., Park M.J. (2011). Morphology and Conductivity in Ionic Liquid Incorporated Sulfonated Block Copolymers. Macromolecules.

[62] Cheng G., Wang Y., Lu Z., Shi J., Chen J., Tian W., Zhu Y., He D., Wu J., Wang H. (2026). Phase separation enables dual ionic and thermal transport in solid-state cathodes. InfoMat.

[63] Schorr N.B., Bhandarkar A., McBrayer J.D., Talin A.A. (2024). Composite Ionogel Electrodes for Polymeric Solid-State Li-Ion Batteries. Polymers.

[64] Li T., Hu A., Li Y., Yang B., Li K., Chen K., Jiang J., Li F., Seh Z.W., Wang J. (2025). Multifunctional Polyfluoride Ionogel-Encapsulated Lithium Anodes for Durable and Safe Pouch Cells under Harsh Conditions. Adv. Funct. Mater..

[65] Chen T., Kong W., Zhang Z., Wang L., Hu Y., Zhu G., Chen R., Ma L., Yan W., Wang Y. (2018). Ionic liquid-immobilized polymer gel electrolyte with self-healing capability, high ionic conductivity and heat resistance for dendrite-free lithium metal batteries. Nano Energy.

[66] Ham S.-Y., Yang H., Nunez-Cuacuas O., Tan D.H., Chen Y.-T., Deysher G., Cronk A., Ridley P., Doux J.-M., Wu E.A. (2023). Assessing the critical current density of all-solid-state Li metal symmetric and full cells. Energy Storage Mater..

[67] Randau S., Weber D.A., Kötz O., Koerver R., Braun P., Weber A., Ivers-Tiffée E., Adermann T., Kulisch J., Zeier W.G. (2020). Benchmarking the performance of all-solid-state lithium batteries. Nat. Energy.

[68] Fawdon J., Ihli J., La Mantia F., Pasta M. (2021). Characterising lithium-ion electrolytes via operando Raman microspectroscopy. Nat. Commun..

[69] Drummond R., Tredenick E.C., Kirk T.L., Forghani M., Grant P.S., Duncan S.R. (2025). Graded Lithium-Ion Battery Pouch Cells to Homogenise Current Distributions and Reduce Lithium Plating. J. Electrochem. Soc..

[70] Taylor M.E., Clarkson D., Greenbaum S.G., Panzer M.J. (2021). Examining the Impact of Polyzwitterion Chemistry on Lithium Ion Transport in Ionogel Electrolytes. ACS Appl. Polym. Mater..

[71] Evans J., Vincent C.A., Bruce P.G. (1987). Electrochemical measurement of transference numbers in polymer electrolytes. Polymer.

[72] Gouverneur M., Schmidt F., Schönhoff M. (2018). Negative effective Li transference numbers in Li salt/ionic liquid mixtures: Does Li drift in the “Wrong” direction?. Phys. Chem. Chem. Phys..

[73] Smiglak M., Reichert W.M., Holbrey J.D., Wilkes J.S., Sun L., Thrasher J.S., Kirichenko K., Singh S., Katritzky A.R., Rogers R.D. (2006). Combustible ionic liquids by design: Is laboratory safety another ionic liquid myth?. Chem. Commun..

[74] Gao J., Wang C., Han D.-W., Shin D.-M. (2021). Single-ion conducting polymer electrolytes as a key jigsaw piece for next-generation battery applications. Chem. Sci..

[75] Yu Y., Lu F., Sun N., Wu A., Pan W., Zheng L. (2018). Single lithium-ion polymer electrolytes based on poly(ionic liquid)s for lithium-ion batteries. Soft Matter.

